# Distance measures and optimization spaces in quantitative fatty acid signature analysis

**DOI:** 10.1002/ece3.1429

**Published:** 2015-02-24

**Authors:** Jeffrey F Bromaghin, Karyn D Rode, Suzanne M Budge, Gregory W Thiemann

**Affiliations:** 1 U.S. Geological Survey, Alaska Science Center4210 University Drive, Anchorage, Alaska, 99508; 2 Process Engineering and Applied Science, Dalhousie UniversityHalifax, Nova Scotia, B3H 4R2, Canada; 3 Faculty of Environmental Studies, York University4700 Keele St., Toronto, Ontario, M3J 1P3, Canada

**Keywords:** Aitchison, Chukchi Sea, diet composition, gray seal, *Halichoerus grypus*, Kullback–Leibler, polar bear, QFASA, *Ursus maritimus*

## Abstract

Quantitative fatty acid signature analysis has become an important method of diet estimation in ecology, especially marine ecology. Controlled feeding trials to validate the method and estimate the calibration coefficients necessary to account for differential metabolism of individual fatty acids have been conducted with several species from diverse taxa. However, research into potential refinements of the estimation method has been limited. We compared the performance of the original method of estimating diet composition with that of five variants based on different combinations of distance measures and calibration-coefficient transformations between prey and predator fatty acid signature spaces. Fatty acid signatures of pseudopredators were constructed using known diet mixtures of two prey data sets previously used to estimate the diets of polar bears *Ursus maritimus* and gray seals *Halichoerus grypus*, and their diets were then estimated using all six variants. In addition, previously published diets of Chukchi Sea polar bears were re-estimated using all six methods. Our findings reveal that the selection of an estimation method can meaningfully influence estimates of diet composition. Among the pseudopredator results, which allowed evaluation of bias and precision, differences in estimator performance were rarely large, and no one estimator was universally preferred, although estimators based on the Aitchison distance measure tended to have modestly superior properties compared to estimators based on the Kullback–Leibler distance measure. However, greater differences were observed among estimated polar bear diets, most likely due to differential estimator sensitivity to assumption violations. Our results, particularly the polar bear example, suggest that additional research into estimator performance and model diagnostics is warranted.

## Introduction

Quantitative fatty acid signature analysis (QFASA) is a method of diet estimation that has gained widespread use since its introduction (Iverson et al. 2004). QFASA has become especially common in investigations of the diets of marine species, presumably because of the importance of lipids as an energy source in marine food webs and the diversity of fatty acids in marine ecosystems (Thiemann et al. 2007). The diets of numerous marine species, including several species of seabirds (Iverson et al. 2007; Williams et al. 2009; Wang et al. 2010) and pinnipeds (Iverson et al. 2004; Beck et al. 2007a,b; Meynier et al. 2010; Bromaghin et al. 2013; Chambellant et al. 2013), as well as polar bears *Ursus maritimus* (Thiemann et al. 2008; Rode et al. 2014) have been estimated using QFASA.

QFASA requires data on the fatty acid signatures (FASs, compositional proportions that sum to 1.0) of one or more predators and all potential prey species, as well as estimates of calibration coefficients (CCs). The CCs are constants used to transform FASs to account for the differential metabolism of individual fatty acids (FAs) and must be estimated via controlled feeding studies (Iverson et al. 2004; Nordstrom et al. 2008; Wang et al. 2010; Budge et al. 2012). Given those inputs, QFASA models a predator FAS as a linear mixture of the mean FASs among prey types. The proportions of predator FAs attributable to each prey type (diet composition) are estimated by minimizing a measure of distance between the observed and modeled predator FASs (Iverson et al. 2004).

Although a growing collection of work has investigated the performance of QFASA and the estimation of CCs for several species from diverse taxa (e.g., Bowen and Iverson 2013), less research into potential refinements of modeling and estimation methods has been conducted. However, variations of the original method have recently been published (Meynier et al. 2010; Stewart and Field 2011; Bromaghin et al. 2013). Iverson et al. (2004) used CCs to transform a predator FAS to the prey FAS space, adjusting for differential FA metabolism, and estimated diet in the prey space. Bromaghin et al. (2013) implemented the alternative approach, transforming mean prey FASs and estimating diet in the predator space. Because diets are estimated by minimizing a distance measure in either the prey or predator FAS space, we refer to them collectively as optimization spaces. There does not seem to be any a priori reason to suspect estimation in either space is consistently superior. However, diet estimates can be expected to differ because the CC transformation alters the magnitudes of the FAS proportions and thereby influences the distance measure being minimized (Budge et al. 2012). In addition, Iverson et al. (2004) based estimation on the Kullback–Leibler (KL) distance measure, as have most subsequent applications of QFASA. One notable exception is Stewart and Field (2011), who minimized the Aitchison (A) distance measure common in analyses of compositional data (Bacon-Shone 2011).

**Figure 1:**
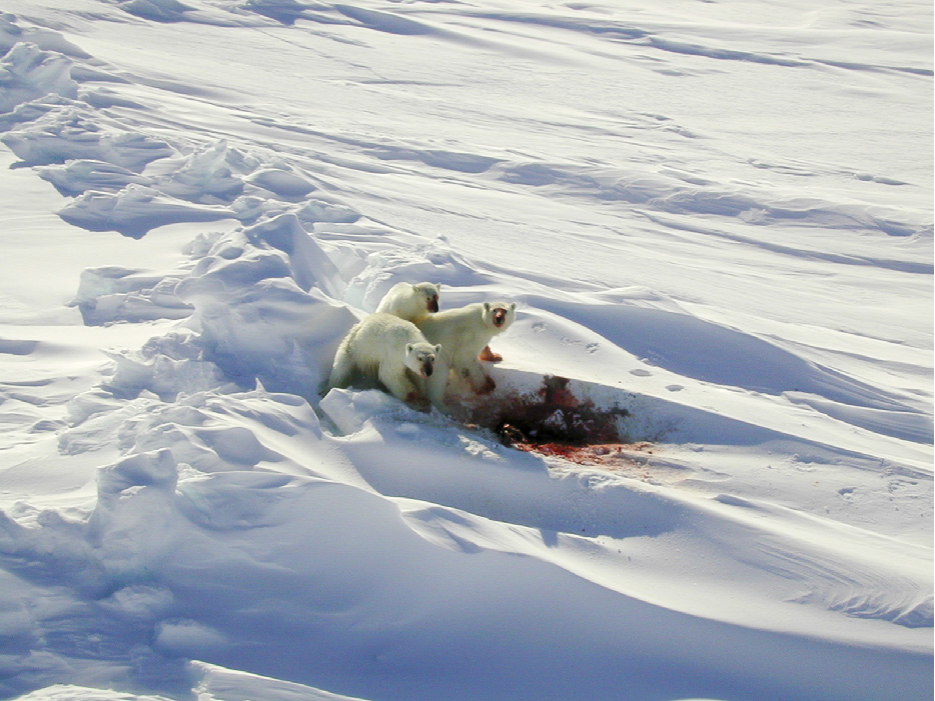
A polar bear (*Ursus maritimus*) family feeding on a ringed seal (*Phoca hispida*). Photograph credit: U.S. Geological Survey.

We used computer simulation to investigate the degree to which the selection of a distance measure (KL or A) and an optimization space (prey or predator) influence QFASA estimates of diet composition. Because CCs provide a one-to-one mapping between the optimization spaces and one would ideally wish to closely approximate the observed FAS in either space, we also investigated the potential benefits of minimizing each distance measure simultaneously in both prey and predator spaces. We expected simultaneous optimization in both spaces to be a stabilizing influence that would reduce the variance of diet estimates. Simulations were conducted with two prey data sets to reduce the possibility that our conclusions would be overly specific to a single data set. We also investigated the influence of distance measures and optimization spaces on estimated diets of Chukchi Sea polar bears (Fig.1), whose diets were previously estimated by Rode et al. (2014).

## Materials and Methods

### Notation and definitions

Our notation closely follows that of Iverson et al. (2004), with slight modification as necessary. Let

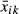
 = the mean proportion of FA k in the FAS of prey type *i*; the *k*^th^ component of the FAS for prey type *i* (prey space),
*c*_*k*_ = the CC for fatty acid *k*,
*π*_*i*_ = the proportion of prey type *i* in the predator diet, and
*y*_*k*_ = the mean proportion of FA *k* in the predator's FAS; the *k*^th^ component of the predator FAS (predator space).
The components of FASs, 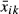 and *y*_*k*_, must sum to unity across FAs. Iverson et al. (2004) used CCs to transform a predator FAS to the prey space, that is,

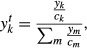
1where the “*t*” superscript denotes a FAS that has been transformed to the alternate space and the summation in the denominator is over all FAs, while Bromaghin et al. (2013) transformed the mean prey FASs to the predator space


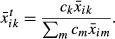
2

In both cases, use of the CCs in the numerator adjusts the magnitudes of the FA proportions to account for a predator's differential metabolism of individual FAs and the denominator rescales the adjusted proportions to sum to unity.

### Distance measures and optimization spaces

Six variants of the QFASA model were constructed from the combinations of two distance measures (KL and A) and three optimizations spaces (prey, predator, and simultaneous prey–predator).

Kullback–Leibler

Operating in the prey space, a transformed predator FAS is modeled as a linear mixture of the mean prey FASs, 

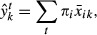
3and the KL distance measure 

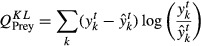
4is minimized with respect to the proportions (*π*_*i*_) to estimate diet (Iverson et al. 2004). The KL distance measure represents a balance between absolute (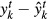) and relative (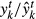) differences between observed and modeled FAS proportions.

In the predator space, a predator FAS is modeled as a linear mixture of the transformed mean prey FASs 

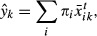
5and the KL distance measure 

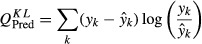
6is similarly minimized with respect to the proportions (*π*_*i*_) to estimate diet composition (Bromaghin et al. 2013).

In addition to the above two variants of the KL objective function (eq. 4 and 6), we investigated the properties of the diet estimator based on simultaneous optimization in both prey and predator spaces by minimizing the objective function 

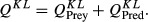
7Aitchison

The A distance measure is based on differences between the ratios of observed and modeled FASs and their respective geometric means (Stewart and Field 2011). The three objective functions (eq. 4, 6, and 7) were modified by replacing the KL distance measure with the A distance measure, that is, 

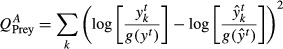
8

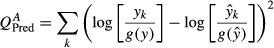
9

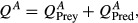
10where *g*(*p*) denotes the geometric mean of a vector of proportions *p*.

### Prey data, fatty acids, and calibration coefficients

Two prey data sets with different characteristics were used to compare diet estimates obtained by minimizing the six objective functions defined above. The first was a marine mammal data set, consisting of FASs from 357 individual prey animals representing seven species (Table S1), previously used to estimate the diets of polar bears from the Chukchi Sea (Rode et al. 2014). The second was a larger and structurally more complex data set of Atlantic fish and shellfish species, consisting of FASs from 957 specimens representing 29 species (Table S2), previously used to investigate the diets of gray seals *Halichoerus grypus* from Sable Island, Nova Scotia, Canada (Budge et al. 2002; Beck et al. 2007a).

We adopted the 31 FAs previously utilized to estimate polar bear diets with the marine mammal prey data (Thiemann et al. 2008; Rode et al. 2014) [16:2n6, 16:2n4, 16:3n6, 16:3n4, 16:4n3, 16:4n1, 18:2n6, 18:3n6, 18:3n4, 18:3n3, 18:3n1, 18:4n3, 18:4n1, 20:1n11, 20:1n9, 20:1n7, 20:2n6, 20:3n6, 20:4n6, 20:3n3, 20:4n3, 20:5n3, 22:1n11, 22:1n9, 22:1n7, 21:5n3, 22:4n6, 22:5n6, 22:4n3, 22:5n3, and 22:6n3] and the “all mink” CCs of Thiemann et al. (2008) for use in pseudopredator simulations with both prey data sets. We did not attempt to independently evaluate the suitability or sufficiency of these FAs or CCs for use with either data set, although they have been used in polar bear applications (Thiemann et al. 2008; Rode et al. 2014) and Beck et al. (2007a) used all but two of these FAs (16:4n3 and 18:3n1, plus ten more). Because pseudopredator FASs were constructed directly from prey data (below), rather than being obtained from biological samples, use of these FAs and CCs in the simulations was largely inconsequential.

### Predator diets and fatty acid signatures

We implemented two different strategies, “random” and “realistic”, for establishing predator diets (*π*_*i*_). In the random diets strategy, sets of diet proportions, non-negative and summing to 1.0, were generated purely at random. This allowed us to compare the performance of the six QFASA variants across the range of all diets possible with each prey data set. We hypothesized that diet diversity might influence estimator performance and therefore computed an order two diversity measure (Leinster and Cobbold 2012) for each random diet. The similarity matrices used to compute diversity, one for each prey data set, were constructed using a leave-one-out algorithm. For each data set, an individual prey FAS was removed from the data and the diet of that individual was estimated as if it were a predator, with CCs set to 1.0. The degree to which the estimated diet was concentrated on the correct prey type, or was dispersed among incorrect prey types, was taken as a measure of similarity among prey types. This process was repeated for each prey FAS in the data set, and the mean estimated diets by prey type were used to construct a similarity matrix. Such a similarity matrix was constructed using each of the QFASA variants, and their average was the similarity matrix used to compute diet diversity.

In the realistic diets strategy, predator diets were based on published diet estimates of specific classes of predators, with some elimination of prey with minor contributions and rounding to convenient values. For the marine mammal prey data set, we established diets for adult female and adult male polar bears (Table S1) based on mean diet estimates reported by Rode et al. (2014). For the marine fish and shellfish prey data set, diets for female and male gray seals captured in both the spring and fall (Table S2) were based on mean diet estimates of Beck et al. (2007a). This strategy allowed us to compare the performance of the six QFASA variants for diets that were known to be realistic for specific classes of predators.

A bootstrapping procedure was used to incorporate random variation in predator FASs. For a given predator diet, either random or realistic, a bootstrap sample (sampling with replacement) of prey FASs was drawn, with sample sizes for each prey type equal to the actual sample sizes in the prey data. The mean FAS of each prey type was computed from the bootstrap sample. There are two possible methods of constructing a predator FAS from such a bootstrap sample. A predator FAS could be computed as a linear mixture of the bootstrapped mean prey FASs using equation 3, after which it would be transformed to the predator space using equation 2. Alternatively, a predator FAS could be constructed by transforming the bootstrapped mean prey FASs to the predator space using equation 1 and then computing the linear mixture using equation 5. Because these two methods produce very similar, but not identical, predator FASs, we made both computations and established a predator FAS as the average of the two to avoid the possibility that the method of constructing a predator FAS might favor one QFASA variant over another. An independent bootstrap sample of prey FASs was drawn for each predator diet.

### Simulation methods

With the marine mammal prey data, 10,000 diets were randomly generated. For each of these diets, 100 pseudopredator FASs were independently established as previously described. Diet composition was estimated for each of the 100 predators. The mean absolute value of the bias (bias) between estimated and true diet proportions 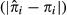 was summed across all diet proportions within each predator and then averaged over the 100 predators, producing a single measure of bias for each random diet. The variance of the estimates of each diet component was computed across the 100 predators sharing a diet and then averaged across diet components (prey types), also producing a single measure of variance for each random diet. For each diet, the bias and variance measures were combined via computation of the root mean squared error (RMSE) as the square root of the sum of the variance and the squared bias.

Identical methods were used to create random diets from the marine fish and shellfish prey data. However, the increased computational time required by the larger data set caused us to reduce the number of random diets to 5000 and the number of predators per diet to 50.

Similar methods were used for the realistic diets (Tables S1, S2). However, in this case only six diets were considered, two polar bear and four gray seal diets, and summary statistics were not computed across diets. We generated 500 pseudopredator FASs for each of these six diets and computed measures of bias, variance, and RMSE for each diet as described above.

### Chukchi Sea polar bear diets

The random and realistic diet simulations were constructed so that the assumptions of the mixture model were perfectly satisfied, and the measures of performance (bias and RMSE) would therefore summarize the performance of the QFASA variants under ideal conditions. However, estimator performance under ideal conditions is not necessarily indicative of performance with biological samples, which may incorporate the effects of assumption violations. We therefore, as an example, replicated previously published diet estimates for adult male, adult female, subadult male, and subadult female polar bears from the Chukchi Sea (Rode et al. 2014) using all six QFASA variants. The standard error of the mean estimated diet composition for each sex and age class combination was estimated using the estimator of Beck et al. (2007a), with 500 bootstrap replications for each bear.

### Diet estimation

The random and realistic diet simulations were implemented using a custom computer program written in Fortran (Metcalf et al. 2008) and compiled to an executable (exe) file. Estimates of Chukchi Sea polar bear diets were obtained using a subset of the Fortran code compiled to a dynamic-link library (dll) file called from scripts written in R version 3.1.1 (R Core Team 2014). In both cases, objective functions were minimized using a reduced gradient algorithm with a golden ratio line search (Luenberger 1984), and Fortran code was compiled with the Intel Visual Fortran Composer XE 2015 compiler (http://software.intel.com/en-us/intel-composer-xe). Neither the KL nor A distance measure is defined for FAS proportions of 0. Consequently, any values of 0 within FASs were replaced with 1.0E-5 and the FAS proportions were rescaled to sum to unity using the multiplicative method (Martín-Fernández et al. 2011).

## Results

### Random diets

**Figure 2:**
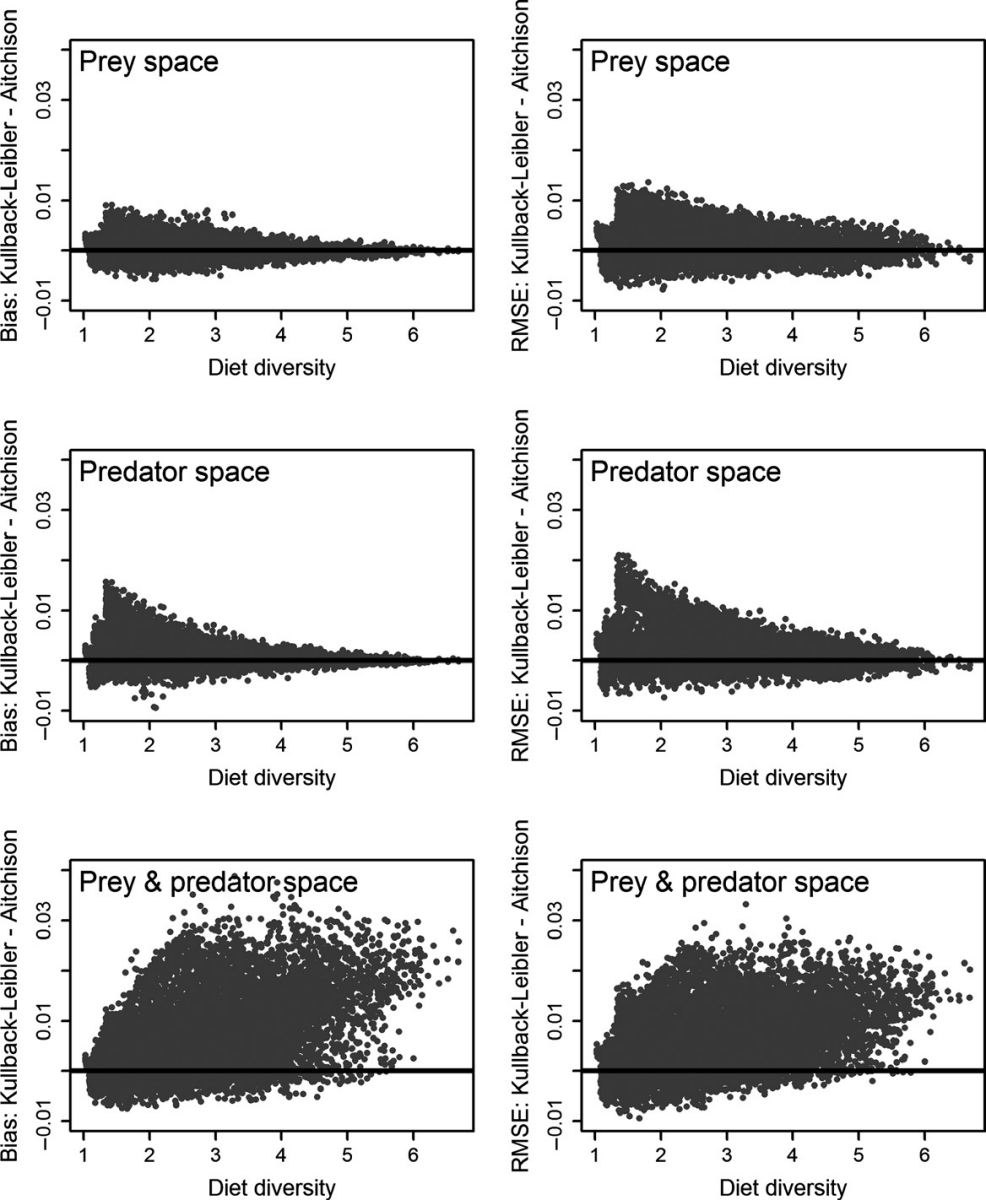
Differences between Kullback–Leibler and Aitchison bias and root mean squared error (RMSE) summary statistics of diet composition estimates as a function of diet diversity based on 10,000 diets randomly generated from the marine mammal data set. The composition of each diet was estimated in the prey, predator, and combined prey–predator spaces.

Estimates of random diets constructed from the marine mammal prey data obtained with the A distance measure tended to have less bias and smaller RMSE than estimates based on the KL distance measure (Table1). When averaged over all 10,000 diets, the A distance measure had 2.4%, 7.9%, and 42.3% less bias and 5.5%, 7.8%, and 21.4% smaller RMSE in the prey, predator, and prey–predator spaces, respectively. Bias and RMSE statistics of diet estimates based on the A distance measure also had smaller standard deviations than the KL statistics (Table1). Bias and RMSE statistics of the two measures were correlated and often similar in the prey and predator spaces, although the performance of the KL measure tended to degrade as diet diversity decreased (Fig.2). The performance of the KL measure was consistently poor in the combined prey–predator space. Overall, the A distance measure minimized in the combined prey–predator optimization space had the least bias and the smallest RMSE (Table1).

**Table 1:** The mean and standard deviation (SD) of mean absolute bias and root mean squared error (RMSE) for each of the six QFASA variants computed across 10,000 diets randomly constructed using the marine mammal prey data set.

Measure	Space	Bias		RMSE	
		Mean	SD	Mean	SD
Kullback–Leibler	Prey	0.0123	0.0060	0.0256	0.0099
Kullback–Leibler	Predator	0.0114	0.0054	0.0258	0.0097
Kullback–Leibler	Both	0.0156	0.0103	0.0285	0.0111
Aitchison	Prey	0.0120	0.0056	0.0242	0.0095
Aitchison	Predator	0.0105	0.0044	0.0238	0.0087
Aitchison	Both	0.0090	0.0048	0.0224	0.0087

**Figure 3:**
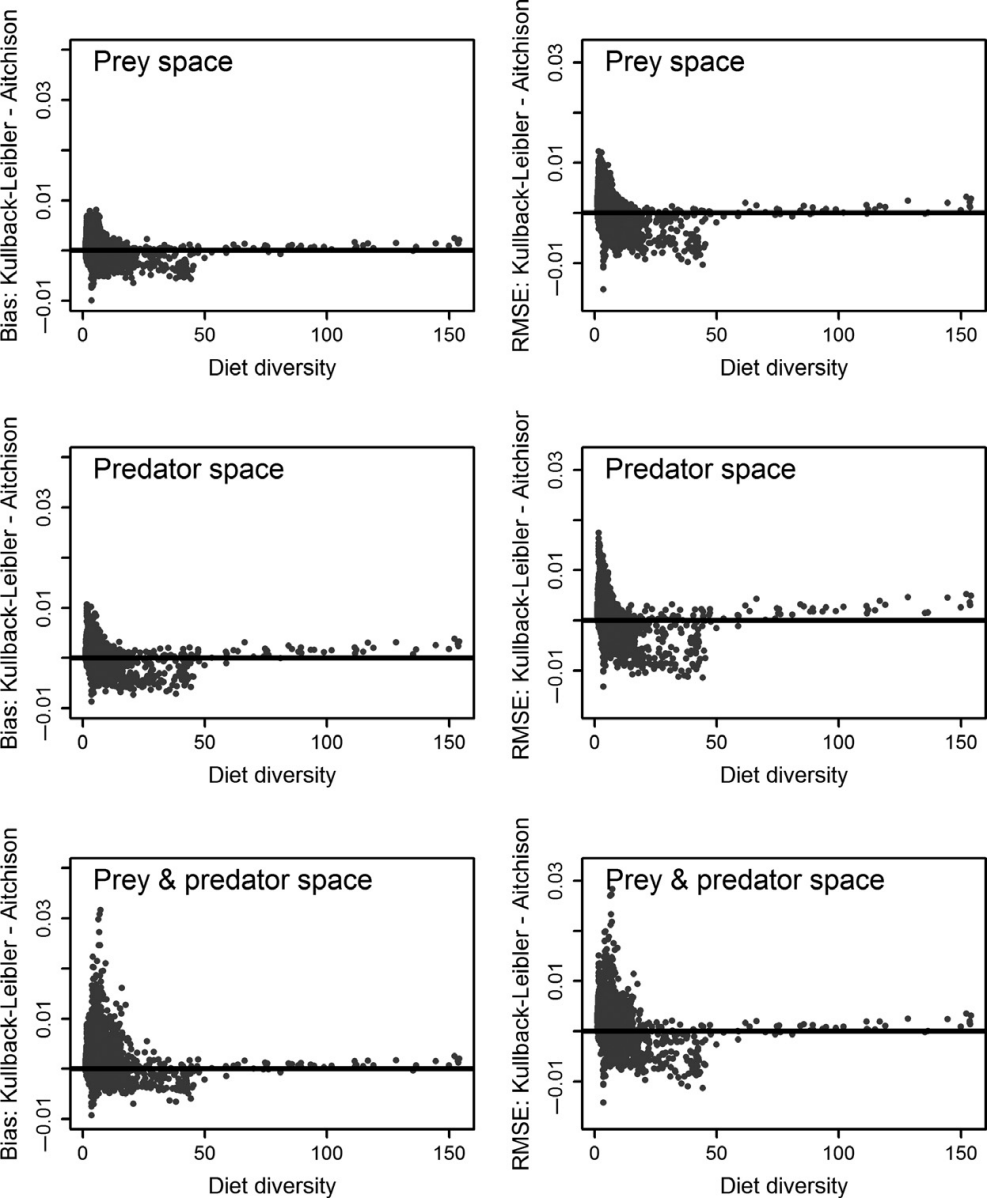
Differences between Kullback–Leibler and Aitchison bias and root mean squared error (RMSE) summary statistics of diet composition estimates as a function of diet diversity based on 5000 diets randomly generated from the fish and shellfish data set. The composition of each diet was estimated in the prey, predator, and combined prey–predator spaces.

Bias and RMSE statistics of diets based on the marine fish and shellfish data set were less divergent than with the marine mammal data (Table2). The KL measure in the combined prey–predator space again had the largest bias and RMSE, but statistics for the other five variants were nearly identical. The KL distance measure again displayed a tendency for increased bias and RMSE with some low-diversity diets, although the A measure tended to have larger bias and RMSE with diets of intermediate diversity (Fig.3, range 25–50).

**Table 2:** The mean and standard deviation (SD) of mean absolute bias and root mean squared error (RMSE) for each of the six QFASA variants computed across 5000 diets randomly constructed using the marine fish and shellfish prey data set.

Measure	Space	Bias		RMSE	
		Mean	SD	Mean	SD
Kullback–Leibler	Prey	0.0091	0.0034	0.0167	0.0051
Kullback–Leibler	Predator	0.0091	0.0034	0.0168	0.0050
Kullback–Leibler	Both	0.0105	0.0048	0.0178	0.0058
Aitchison	Prey	0.0089	0.0032	0.0167	0.0053
Aitchison	Predator	0.0089	0.0033	0.0167	0.0054
Aitchison	Both	0.0091	0.0034	0.0168	0.0054

We were able to identify certain diet combinations for which one distance measure tended to perform better than the other, although the tendencies were not universally consistent. With the marine mammal prey data set, QFASA variants based on the KL distance measure tended to have lower bias and RMSE for diets dominated by bowhead whale *Balaena mysticetus*; beluga whale *Delphinapterus leucas*; ribbon seal *Histriophoca fasciata*; and spotted seal *Phoca largha*. Conversely, estimators based on the A distance measure tended to have superior properties with diets dominated by ringed seal *Pusa hispida*; bearded seal *Erignathus barbatus* and beluga whale; and a mixture of beluga whale, ribbon seal, and spotted seal. Similar patterns were apparent in estimated diets from the fish and shellfish data, although they were more complex. The KL measure tended to have lower bias and RMSE with diets comprised of ocean pout *Zoarces americanus* and plaice *Hippoglossoides platessoides* and mixtures of Atlantic argentine *Argentina silus*, white hake *Urophycis tenuis*, winter flounder *Pseudopleuronectes americanus*, winter skate *Leucoraja ocellata*, and yellowtail *Limanda ferruginea*. Conversely, the A distance measure tended to have superior performance with diets comprised of mixtures of sea raven *Hemitripterus americanus*, silver hake *Merluccius bilinearis*, and smooth skate *Malacoraja senta*; Atlantic argentine, butterfish *Peprilus triacanthus*, capelin *Mallotus villosus*, cod *Gadus morhua*, and yellowtail; and red crab *Chaceon quinquedens*, red fish *Sebastes fasciatus*, rock crab *Urophycis chuss*, sea raven, and silver hake. We were, however, unable to identify specific aspects of either diet composition or the data underlying these patterns, and suspect they were caused by a complex interaction of the diet proportions, prey FASs, and differences in the how the KL and A measures quantify distance between observed and modeled predator FASs.

### Realistic diets

The A distance measure in the prey space produced estimates of the realistic diet for adult male polar bears with the smallest bias and RMSE statistics (Table3). Although KL estimates in the prey space had slightly less bias for the largest diet component, ringed seal, they also had greater variance, and the A distance measure had both less bias and smaller RMSE statistics for most diet components. Overall, estimates based on the A distance measure had 7.8%, 16.3%, and 18.1% less bias and 24.8%, 23.2%, and 26.9% smaller RMSE in the prey, predator, and combined prey–predator spaces, roughly comparable to the values observed with the random diets. However, the performance of the KL measure in the combined prey–predator space was not notably worse than that of the other variants. Estimates based on the combined prey–predator space tended to be intermediate between the other estimates in terms of bias, which we anticipated, but also in terms of RMSE, which was not expected. The results for adult female polar bears were similar (Table S3).

**Table 3:** Bias and root mean squared error (RMSE) of diet estimates for adult male polar bears *Ursus maritimus* generated by each of the six QFASA variants, based on the marine mammal prey data set and computed across 500 randomly generated FASs.

		Kullback–Leibler						Aitchison					
		Prey space		Pred. space		Both spaces		Prey space		Pred. space		Both spaces	
Prey species	Truediet	Bias	RMSE	Bias	RMSE	Bias	RMSE	Bias	RMSE	Bias	RMSE	Bias	RMSE
*Erignathus barbatus*	0.207	−0.003	0.056	−0.014	0.058	−0.007	0.056	−0.002	0.031	−0.004	0.031	−0.003	0.031
*Delphinapterus leucas*	0.014	−0.009	0.013	−0.010	0.013	−0.013	0.014	−0.007	0.011	−0.004	0.014	−0.009	0.012
*Balaena mysticetus*	0.077	−0.007	0.021	0.005	0.025	−0.000	0.022	−0.006	0.020	0.004	0.022	0.001	0.020
*Histriophoca fasciata*	0.000	0.007	0.016	0.009	0.023	0.008	0.019	0.010	0.024	0.015	0.036	0.011	0.027
*Pusa hispida*	0.658	−0.006	0.060	−0.022	0.076	−0.016	0.069	−0.008	0.044	−0.030	0.058	−0.018	0.049
*Phoca largha*	0.000	0.017	0.037	0.025	0.052	0.025	0.049	0.012	0.026	0.016	0.036	0.015	0.032
*Odobenus rosmarus*	0.044	0.001	0.020	0.007	0.023	0.003	0.021	0.002	0.010	0.003	0.011	0.002	0.011
Sum absolute values	1.000	0.051	0.222	0.092	0.271	0.072	0.249	0.047	0.167	0.077	0.208	0.059	0.182

Patterns in the estimates of realistic gray seal diets among the six QFASA variants were less consistent than in the polar bear estimates. QFASA variants based on the A distance measure were less biased overall, but the optimization space with the least bias differed among the diets. The A measure in the predator space had the smallest bias for spring males (Table4), spring females (Table S4), and fall males (Table S6), although the A measure in the combined prey–predator space had the least bias for fall females (Table S5). However, the RMSE statistics tended to be similar among QFASA variants, except for the KL estimates in the combined prey- predator space, indicating that the A estimates were somewhat more variable. Estimates with the smallest RMSE were either KL estimates in the predator space (Tables S4, S5) or A estimates in the predator space (Table4, Table S6). Similar to our findings with the random diets from both prey data sets, though not the realistic polar bear diets, the KL distance measure performed less well in the combined prey–predator space for all four gray seal diets (Table4, Tables S4–S6). Also, unlike the realistic polar bear diets, the KL estimates for the largest diet components were not consistently less biased than A estimates.

**Table 4:** Bias and root mean squared error (RMSE) of diet estimates for male gray seals *Halichoerus grypus* in the spring generated by each of the six QFASA variants, based on the marine fish and shellfish prey data set and computed across 500 randomly generated FASs.

		Kullback–Leibler						Aitchison					
		Prey space		Pred. space		Both spaces		Prey space		Pred. space		Both spaces	
Prey species	Truediet	Bias	RMSE	Bias	RMSE	Bias	RMSE	Bias	RMSE	Bias	RMSE	Bias	RMSE
*Mallotus villosus*	0.004	0.013	0.022	0.021	0.035	0.066	0.073	0.009	0.018	0.010	0.020	0.017	0.025
*Gadus morhua*	0.039	0.002	0.034	−0.003	0.032	0.000	0.037	0.001	0.033	−0.005	0.029	0.003	0.033
*Clupea harengus*	0.032	−0.011	0.024	−0.010	0.025	−0.031	0.032	−0.001	0.025	0.002	0.027	−0.001	0.026
*Myoxocephalus octodecemspinosus*	0.024	−0.008	0.019	−0.010	0.019	−0.022	0.023	−0.004	0.019	−0.007	0.017	−0.010	0.018
*Ammodytes dubius*	0.071	−0.001	0.028	−0.007	0.028	0.028	0.041	−0.003	0.036	−0.008	0.035	0.008	0.034
*Pollachius pollachius*	0.306	0.007	0.033	−0.022	0.037	−0.078	0.084	0.003	0.031	−0.026	0.038	−0.027	0.040
*Sebastes fasciatus*	0.335	−0.045	0.051	0.016	0.034	−0.082	0.087	−0.042	0.047	0.023	0.033	−0.027	0.036
*Merluccius bilinearis*	0.018	0.008	0.027	0.005	0.026	0.101	0.109	0.005	0.025	0.004	0.024	0.019	0.033
*Amblyraja radiata*	0.033	−0.009	0.017	−0.012	0.017	−0.022	0.024	−0.005	0.016	−0.008	0.016	−0.004	0.013
*Urophycis tenuis*	0.063	0.004	0.022	−0.003	0.017	0.007	0.021	−0.012	0.022	−0.017	0.024	−0.010	0.020
*Pseudopleuronectes americanus*	0.046	−0.001	0.014	−0.007	0.015	0.008	0.013	0.003	0.018	−0.005	0.015	0.001	0.015
*Leucoraja ocellata*	0.006	−0.001	0.010	−0.002	0.008	−0.006	0.006	−0.004	0.007	−0.004	0.007	−0.005	0.006
*Limanda ferruginea*	0.023	−0.003	0.022	−0.008	0.019	−0.023	0.023	−0.001	0.019	−0.005	0.015	−0.008	0.017
Others combined	0.000	0.045	0.052	0.043	0.051	0.053	0.058	0.051	0.057	0.046	0.051	0.045	0.050
Sum absolute values	1.000	0.157	0.375	0.169	0.363	0.528	0.631	0.142	0.372	0.170	0.353	0.185	0.367

### Chukchi Sea polar bear diets

We found greater differences among mean diet estimates of Chukchi Sea polar bears than was observed in either the random or realistic diet simulations (Table5), with the greatest differences occurring among the primary diet components, ringed and bearded seals. The greatest absolute difference in estimated diet components involved the ringed seal contribution to the mean diet of adult males, a difference of 35% between the KL (82%) and A (47%) estimates obtained in the predator space. The maximum difference among the estimated contributions of ringed seals was also at least 14% for adult female and subadult bears, with the A estimates consistently lower than KL estimates. Correspondingly, the A estimates of bearded seal contributions to diet were consistently larger than KL estimates, with the maximum difference among QFASA variants ranging from 9% to 34% among sex and age classes. The estimated contributions of other species to polar bear diets were smaller and more consistent among the QFASA variants.

**Table 5:** Estimated diet composition (percentages) of Chukchi Sea polar bears *Ursus maritimus* by age and sex class for each of the six variants of the QFASA model (mean ± standard error). The mean diets of these bears were previously reported by Rode et al. (2014).

Age–sex/prey species	Kullback–Leibler			Aitchison		
	Prey	Predator	Both	Prey	Predator	Both
Adult males (*n* = 61)						
*Erignathus barbatus*	21 ± 3	7 ± 3	15 ± 3	41 ± 3	40 ± 3	40 ± 3
*Delphinapterus leucas*	1 ± 0	0 ± 0	0 ± 0	4 ± 1	5 ± 1	4 ± 1
*Balaena mysticetus*	3 ± 1	3 ± 1	3 ± 1	7 ± 1	8 ± 1	8 ± 1
*Histriophoca fasciata*	0 ± 0	0 ± 0	0 ± 0	0 ± 0	0 ± 0	0 ± 0
*Pusa hispida*	73 ± 3	82 ± 2	77 ± 3	48 ± 4	47 ± 4	47 ± 4
*Phoca largha*	0 ± 0	0 ± 0	0 ± 0	0 ± 0	0 ± 0	0 ± 0
*Odobenus rosmarus*	2 ± 1	8 ± 1	4 ± 1	0 ± 0	0 ± 0	0 ± 0
Adult females (*n* = 55)						
*Erignathus barbatus*	7 ± 2	1 ± 1	4 ± 1	13 ± 2	13 ± 2	13 ± 2
*Delphinapterus leucas*	0 ± 0	0 ± 0	0 ± 0	2 ± 1	2 ± 1	2 ± 1
*Balaena mysticetus*	1 ± 1	1 ± 1	2 ± 1	5 ± 1	6 ± 1	6 ± 1
*Histriophoca fasciata*	0 ± 0	0 ± 0	0 ± 0	0 ± 0	0 ± 0	0 ± 0
*Pusa hispida*	91 ± 2	96 ± 1	94 ± 1	80 ± 2	79 ± 2	79 ± 2
*Phoca largha*	0 ± 0	0 ± 0	0 ± 0	0 ± 0	0 ± 0	0 ± 0
*Odobenus rosmarus*	0 ± 0	2 ± 0	1 ± 0	0 ± 0	0 ± 0	0 ± 0
Subadult males (*n* = 25)						
*Erignathus barbatus*	7 ± 2	1 ± 1	3 ± 2	10 ± 3	10 ± 2	10 ± 3
*Delphinapterus leucas*	2 ± 2	0 ± 0	0 ± 0	3 ± 2	4 ± 2	3 ± 2
*Balaena mysticetus*	1 ± 1	3 ± 2	3 ± 2	4 ± 1	5 ± 1	5 ± 1
*Histriophoca fasciata*	0 ± 0	0 ± 0	0 ± 0	0 ± 0	0 ± 0	0 ± 0
*Pusa hispida*	89 ± 2	95 ± 2	94 ± 2	81 ± 5	79 ± 5	80 ± 5
*Phoca largha*	0 ± 0	0 ± 0	0 ± 0	2 ± 2	2 ± 2	2 ± 2
*Odobenus rosmarus*	0 ± 0	1 ± 0	0 ± 0	0 ± 0	0 ± 0	0 ± 0
Subadult females (*n* = 13)						
*Erignathus barbatus*	9 ± 4	2 ± 3	4 ± 4	12 ± 6	12 ± 6	12 ± 6
*Delphinapterus leucas*	0 ± 1	0 ± 0	0 ± 0	2 ± 1	2 ± 1	1 ± 1
*Balaena mysticetus*	0 ± 0	0 ± 1	1 ± 1	4 ± 2	4 ± 2	4 ± 2
*Histriophoca fasciata*	0 ± 0	0 ± 0	0 ± 0	0 ± 0	0 ± 0	0 ± 0
*Pusa hispida*	91 ± 4	96 ± 3	95 ± 4	83 ± 7	82 ± 7	83 ± 7
*Phoca largha*	0 ± 0	0 ± 0	0 ± 0	0 ± 1	0 ± 1	0 ± 1
*Odobenus rosmarus*	0 ± 0	1 ± 1	1 ± 1	0 ± 0	0 ± 0	0 ± 0

KL estimates were more variable among the optimization spaces than the A estimates, as we observed in the simulation results. None of the A estimates differed by more than 2%, while the KL estimates differed by up to 14%. The A and KL estimates obtained in the prey space were most similar.

The KL estimates in the prey space (Table5) were most similar to the estimates reported by Rode et al. (2014; Table S6), as expected given the similarity in methods. However, the estimates were not identical for potentially four reasons: optimization methods differed; methods of replacing FAS proportions of zero differed; estimates reported by Rode et al. (2014) were the average of estimates generated by two different sets of CCs (Thiemann et al. 2008), whereas we used a single set of CCs; and Rode et al. (2014) reported standard deviations of diet proportions across individual bears, rather than standard errors of estimated means (Beck et al. 2007a).

**Figure 4:**
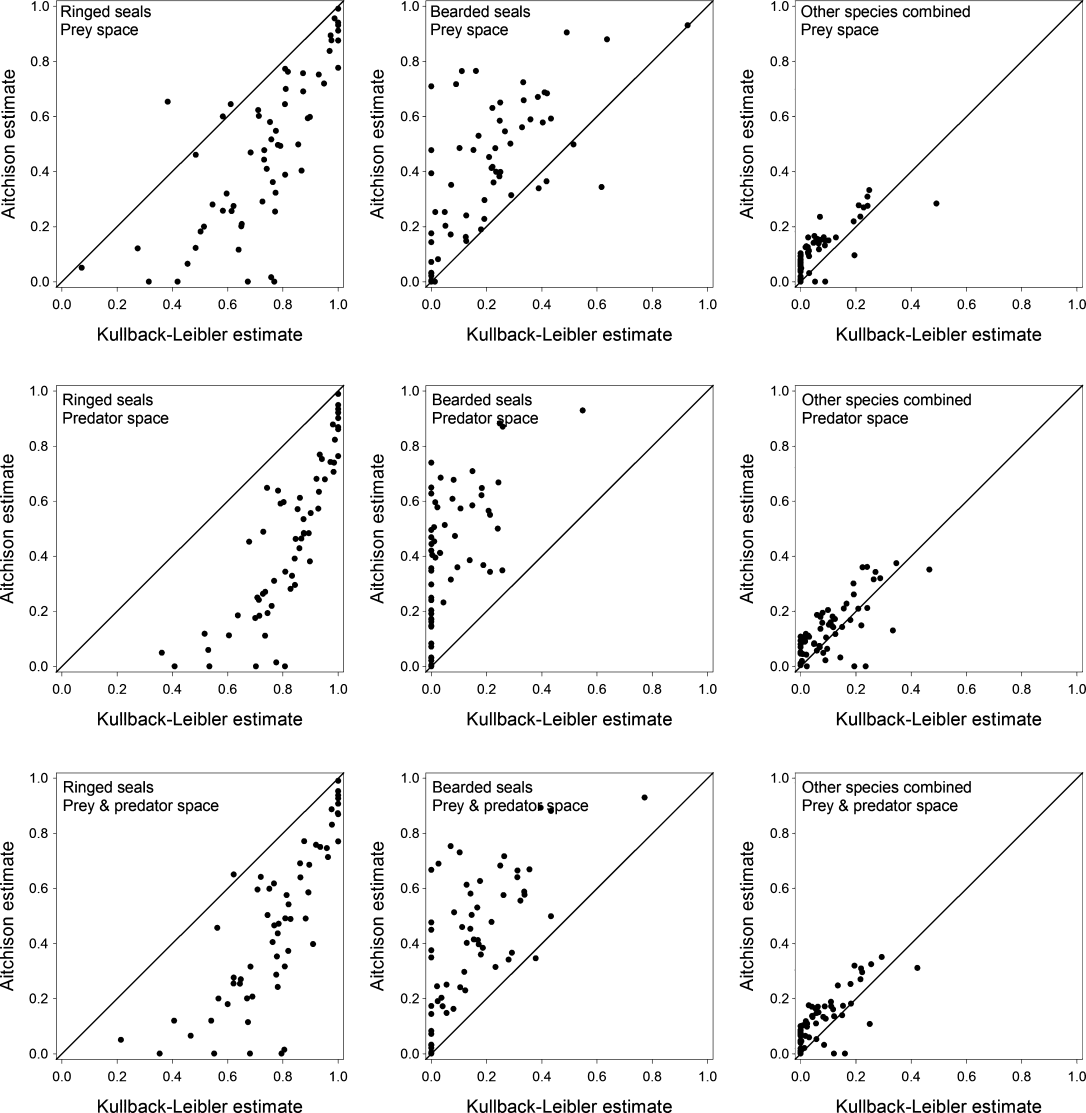
Estimated contributions (proportions) of ringed seal *Pusa hispida*, bearded seal *Erignathus barbatus*, and all other species combined to the diets of individual adult male Chukchi Sea polar bears *Ursus maritimus*. The mean diets of these bears were previously reported by Rode et al. (2014).

Differences among the mean diet estimates (Table5) originate in the estimated diets of individual polar bears. As an example, consider the estimated contributions of bearded seal, ringed seal, and other species combined to the diets of adult males (Fig.4). The A estimates of both ringed and bearded seals were almost uniformly distributed over a broad range of small to large proportions. Conversely, the KL estimates were more concentrated at small proportions for bearded seals and large proportions for ringed seals. The only similarity between the two sets of estimates is that the contributions of species other than bearded and ringed seals were nearly all less than 40%, and most were less than 20%. Similar, but less pronounced, tendencies were observed in the estimates for the other sex and age classes (Figs. S1–S3).

**Figure 5:**
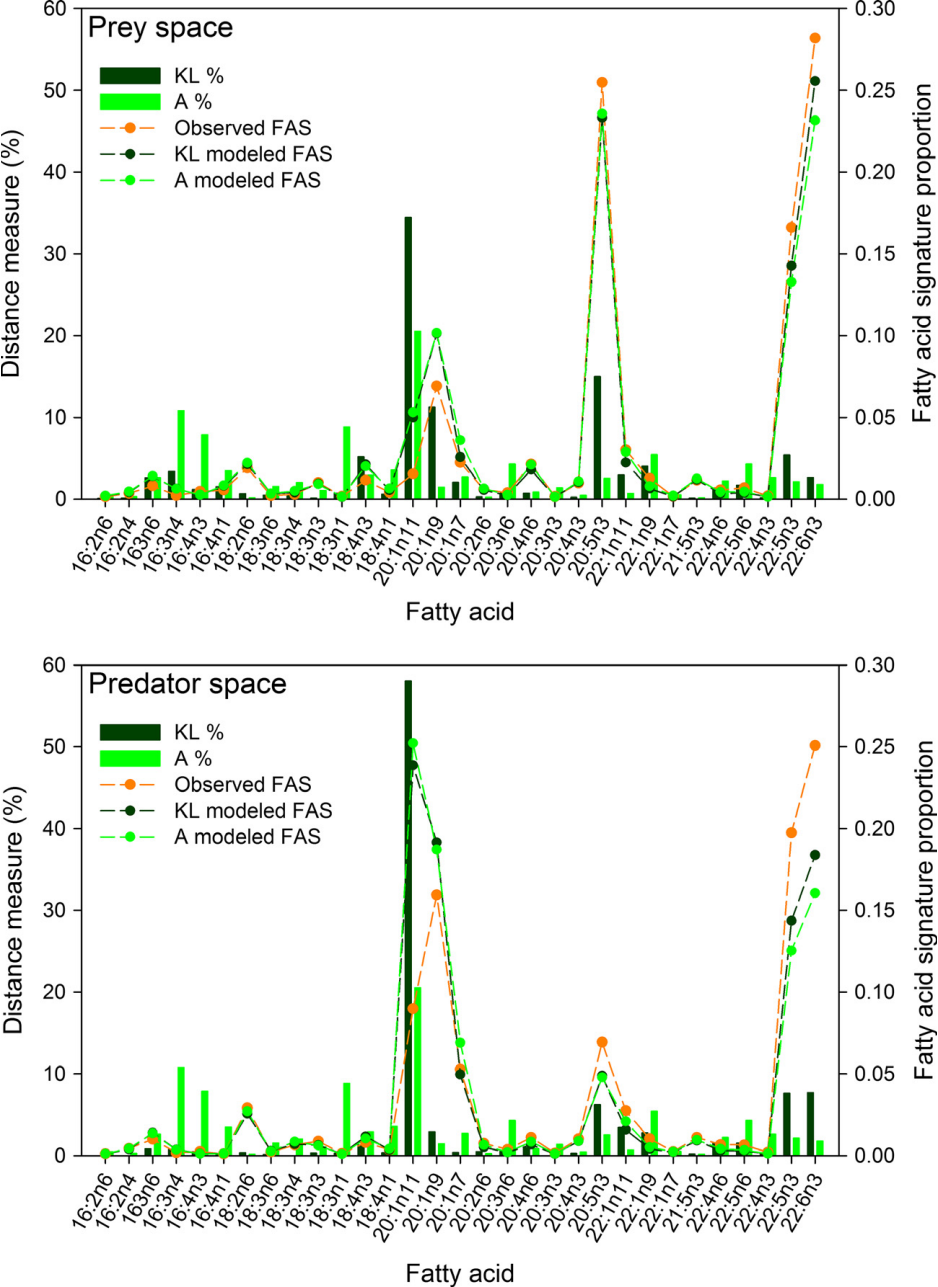
The percentages of the Kullback–Leibler and Aitchison distance measures attributable to each fatty acid (bars) and the observed and modeled fatty acid signatures (lines) in the prey (top panel) and predator (bottom panel) estimation spaces, averaged over all adult male Chukchi Sea polar bears *Ursus maritimus*.

To further explore the differential performance of the KL and A distance measures, we conducted post hoc comparisons of observed and modeled FASs and the proportion of each minimized distance measure attributable to each FA, based on averages computed across bears within each sex and age class. The KL and A distance measures appeared to be sensitive to different aspects of the observed and modeled FASs. A single FA (20:1n11) contributed most to both KL and A distance measures for adult males (Fig.5). Absolute differences between the observed and modeled FAS proportions for this fatty acid were among the largest in both the prey and predator spaces, and the relative differences were also relatively large (over 2). The other FAs with the largest contributions to the KL distance measure tended to have large FAS proportions with relatively large absolute differences and modest relative differences between observed and modeled FAS proportions (e.g., 20:1n9, 20:5n3, 22:5n3, and 22:6n3). Conversely, the other FAs with the largest contributions to the A distance measure tended to have small proportions with large relative and small absolute differences between observed and modeled FAS proportions (e.g., 16:3n4, 16:4n3, 18:3n1, 22:1n9). Similar patterns were observed among the other sex and age classes (Figs. S4–S6). A large proportion of polar bears had FAS proportions outside the range of mean prey proportions for several FAs with large contributions to the distance measures (16:3n4-100%, 16:4n3-100%, 18:3n1-85%, 20:1n11-96%, 20:1n9-57%, 20:5n3-100%). Considering all bears and all FAs, 46% of the FAS proportions were outside the observed range of the mean prey FAS proportions, which undoubtedly reduced the ability of the models to closely approximate the observed FASs and contributed to the absolute and relative differences the distance measures are based on.

## Discussion

The random and realistic diet simulations are informative because they reveal the performance of the QFASA variants under known conditions. However, it is important to recognize that model assumptions were fully satisfied in the simulations, that is, both the CCs and the prey FASs were known perfectly. For that reason, the small mean bias we observed was not surprising, and the results provided additional reassurance that the basic method works well when assumptions are satisfied. The variance of the estimates, which was incorporated into the RMSE statistics, was also small. However, the magnitude of the variance in the simulations was primarily controlled by the sample sizes used while bootstrap resampling prey FASs for construction of predator FASs. Because the bootstrap sample sizes were equal to the sample sizes within the prey data sets, many of which were large, the variation among predator FASs was likely less than would typically be observed with biological samples from free-ranging predators. For these reasons, the relative magnitudes of the bias and RMSE statistics are more informative than their absolute differences.

Patterns and similarities among the random and realistic simulation results with the two data sets are informative with respect to the QFASA variants. One conclusion that can be made with some confidence is to avoid optimization of the KL distance measure in the combined prey–predator space, as that combination tended to produce estimates having the greatest bias and variance with both data sets. With that combination excluded from further consideration, developing recommendations based on the remaining results is more tenuous. Estimators based on the A distance measure tended to have superior properties, being somewhat less biased with both data sets and having competitive or superior RMSE statistics. Although KL and A estimates were correlated, the A estimators did not display the elevated bias and variance that sometimes occurred with the KL estimators, especially with low-diversity diets (Figs.2, 3). In addition, estimators based on the A distance measure were consistently less sensitive to the optimization space used, a stability characteristic which we interpret as advantageous. For those reasons, we would tend to favor the A distance measure, in the absence of any other information, although the performance of estimators based on the KL distance measure in the prey and predator spaces were not so poor that we would advise against their use.

We did not find estimation to be consistently superior in any one optimization space, although estimates for individual diets varied, sometimes substantially, among the spaces. Estimation in the predator space was competitive or superior with the random diets, while both the prey and predator spaces had superior statistics for some of the realistic diets. Estimation in the combined prey–predator space, when using the A distance measure, occasionally had the minimum bias and RMSE. However, estimates in the combined space were more often intermediate between those obtained in the prey and predator spaces, and did not display the reduced variance we anticipated. Based on these results, estimating diet composition in the combined space does not appear to be worth the additional complexity and computational expense, although it perhaps could be viewed as a safeguard against poor performance in a single space.

We observed greater differences between diet estimates based on the KL and A distance measures in the Chukchi Sea polar bear example than was typical in the simulations. Although we were unable to conclusively identify a cause, the data and the analysis provided informative clues. In particular, the large number of FAS proportions that fell outside the range of mean prey proportions, which is conceptually impossible for a linear mixture model, suggests that model assumptions were violated to some extent. One potential violation is that polar bears consumed large quantities of prey that were not represented in the prey data set. Based on extensive field observations and prior investigations of polar bear diets in the Chukchi Sea and the neighboring southern Beaufort Sea (e.g., Bentzen et al. 2007; Thiemann et al. 2008; Cherry et al. 2011; Voorhees et al. 2014), we do not believe a major violation of this assumption is likely. A second potential violation is the existence of inaccuracies in the CCs, which could be attributable to physiological mechanisms that differ between polar bears and mink. Inaccuracies in the CCs could either over- or under-inflate FAS proportions in the transformation between predator and prey spaces, introducing bias to diet estimates and potentially causing predator proportions to fall outside the range of prey proportions.

The polar bear example also revealed important differences in the response of the KL and A distance measures to FAS characteristics. The KL measure is the product of two terms, one that measures the absolute difference between observed and modeled FAS proportions and another that measures the relative difference. Conversely, the A measure is more strongly founded on relative differences. Two small proportions, such as 0.001 and 0.005, may have a small absolute difference, but a large relative difference, while the opposite is more likely for two larger proportions, such as 0.3 and 0.4. For those reasons, differences between observed and modeled proportions may tend to influence the KL measure more when the proportions are large and the A measure when they are small, a tendency we observed in the polar bear example.

In summary, our findings reveal that the selection of an estimation method has the potential to meaningfully influence estimates of diet composition, as can the selection of CCs and FAs (Iverson et al. 2004; Meynier et al. 2010; Wang et al. 2010; Budge et al. 2012). Differences among estimates were at times large enough to be biologically meaningful, in both the simulation results and the polar bear example. However, no one method was found to be consistently superior. For these reasons, the most prudent approach to selecting an estimation method in individual investigations may be to construct a number of plausible diets and compare the performance of candidate estimation methods, essentially replicating the realistic diet component of our work. That approach, unfortunately, increases the complexity and computational burden of an investigation. In addition, the polar bear example suggests that characteristics of predator and prey data may be important determinants of model performance in at least some investigations, in which case such simulations may be less informative than one might normally expect. Additional research into the robustness of estimation methods to violations of model assumptions appears warranted, as does additional development of diagnostic methods for evaluation of model suitability. Further work to evaluate estimator performance under known conditions, perhaps through a re-examination of existing data from controlled feeding trials, may also be informative.

## Supporting Information

**Table S1.** Sample size (*n*) by species in the marine mammal prey data set, along with realistic diet proportions for adult female (AF) and adult male (AM) polar bears *Ursus maritimus* slightly modified from the estimates of Rode et al. (2014). **Table S2.** Sample size (*n*) by species in the marine fish and shellfish prey data set, along with realistic diet proportions for grey seal *Halichoerus grypus* females in spring (SF), males in spring (SM), females in fall (FF), and males in fall (FM) slightly modified from the estimates of Beck et al. (2007a). **Table S3.** Bias and root mean squared error (RMSE) of diet estimates for adult female polar bears *Ursus maritimus* generated by each of the six QFASA variants, based on the marine mammal prey data set and computed across 500 randomly generated FASs. **Table S4.** Bias and root mean squared error (RMSE) of diet estimates for female grey seals *Halichoerus grypus* sampled in the spring generated by each of the six QFASA variants, based on the marine fish and shellfish prey data set and computed across 500 randomly generated FASs. **Table S5.** Bias and root mean squared error (RMSE) of diet estimates for female grey seals *Halichoerus grypus* sampled in the fall generated by each of the six QFASA variants, based on the marine fish and shellfish prey data set and computed across 500 randomly generated FASs. **Table S6.** Bias and root mean squared error (RMSE) of diet estimates for male grey seals *Halichoerus grypus* sampled in the fall generated by each of the six QFASA variants, based on the marine mammal prey data set and computed across 500 randomly generated FASs. **Figure S1.** Estimated contributions (proportions) of ringed seal *Pusa hispida*, bearded seal *Erignathus barbatus*, and all other species combined to the diets of individual adult female Chukchi Sea polar bears *Ursus maritimus*. **Figure S2.** Estimated contributions (proportions) of ringed seal *Pusa hispida*, bearded seal *Erignathus barbatus*, and all other species combined to the diets of individual subadult male Chukchi Sea polar bears *Ursus maritimus*. **Figure S3.** Estimated contributions (proportions) of ringed seal *Pusa hispida*, bearded seal *Erignathus barbatus*, and all other species combined to the diets of individual subadult female Chukchi Sea polar bears *Ursus maritimus*. **Figure S4.** The percentages of the Kullback-Leibler and Aitchison distance measures attributable to each fatty acid (bars) and the observed and modeled fatty acid signatures (lines) in the prey (top panel) and predator (bottom panel) estimation spaces, averaged over all adult female Chukchi Sea polar bears *Ursus maritimus*. **Figure S5.** The percentages of the Kullback-Leibler and Aitchison distance measures attributable to each fatty acid (bars) and the observed and modeled fatty acid signatures (lines) in the prey (top panel) and predator (bottom panel) estimation spaces, averaged over all subadult male Chukchi Sea polar bears *Ursus maritimus*. **Figure S6.** The percentages of the Kullback-Leibler and Aitchison distance measures attributable to each fatty acid (bars) and the observed and modeled fatty acid signatures (lines) in the prey (top panel) and predator (bottom panel) estimation spaces, averaged over all subadult female Chukchi Sea polar bears *Ursus maritimus*.
