# Supplementary material for: Distance measures and optimization spaces in quantitative fatty acid signature analysis
Source: Ecol Evol. 2015 Feb 24;5(6):1249–62. doi: 10.1002/ece3.1429 (PMC4377268; doi:10.1002/ece3.1429)
Supplement: Supplementary file 1 [file ece30005-1249-sd1.docx]

Supplement. Sample size by species for the two prey data sets and the realistic diet compositions explored with each; statistics summarizing diet estimates obtained with the six QFASA variants for adult female polar bears *Ursus maritimus*, and grey seal *Halichoerus grypus* females sampled in the spring and fall and males sampled in the fall; figures showing estimated diet composition for adult female, subadult male, and subadult female polar bears from the Chukchi Sea; and figures showing observed and modeled fatty acid signatures, along with the contributions of individual fatty acids to the Kullback-Leibler and Aitchison distance measures, for adult female, subadult male, and subadult female Chukchi Sea polar bears.

| Table S1. Sample size (n) by species in the marine mammal prey data set, along with realistic diet proportions for adult female (AF) and adult male (AM) polar bears *Ursus maritimus* slightly modified from the estimates of Rode at al. (2014) | | | | | | |
| --- | --- | --- | --- | --- | --- | --- |
| Prey species |  | n |  | AF diet |  | AM diet |
| *Erignathus barbatus* |  | 83 |  | 0.065 |  | 0.207 |
| *Delphinapterus leucas* |  | 29 |  | 0.005 |  | 0.014 |
| *Balaena mysticetus* |  | 64 |  | 0.051 |  | 0.077 |
| *Histriophoca fasciata* |  | 32 |  | 0.000 |  | 0.000 |
| *Pusa hispida* |  | 23 |  | 0.873 |  | 0.658 |
| *Phoca largha* |  | 24 |  | 0.000 |  | 0.000 |
| *Odobenus rosmarus* |  | 102 |  | 0.006 |  | 0.044 |
| Totals |  | 357 |  | 1.000 |  | 1.000 |

| Table S2. Sample size (n) by species in the marine fish and shellfish prey data set, along with realistic diet proportions for grey seal *Halichoerus grypus* females in spring (SF), males in spring (SM), females in fall (FF), and males in fall (FM) slightly modified from the estimates of Beck et al. (2007a) | | | | | | | |
| --- | --- | --- | --- | --- | --- | --- | --- |
| Prey species |  | n |  | SF diet | SM diet | FF diet | FM diet |
| *Argentina silus* |  | 10 |  | 0.000 | 0.000 | 0.000 | 0.000 |
| *Peprilus triacanthus* |  | 10 |  | 0.000 | 0.000 | 0.000 | 0.000 |
| *Mallotus villosus* |  | 56 |  | 0.013 | 0.004 | 0.022 | 0.030 |
| *Gadus morhua* |  | 84 |  | 0.005 | 0.039 | 0.017 | 0.010 |
| *Alosa pseudoharengus* |  | 41 |  | 0.000 | 0.000 | 0.000 | 0.000 |
| *Melanogrammus aeglefinus* |  | 54 |  | 0.000 | 0.000 | 0.000 | 0.000 |
| *Hippoglossus hippoglossus* |  | 8 |  | 0.000 | 0.000 | 0.000 | 0.000 |
| *Clupea harengus* |  | 74 |  | 0.053 | 0.032 | 0.014 | 0.037 |
| *Homarus americanus* |  | 9 |  | 0.000 | 0.000 | 0.000 | 0.000 |
| *Myoxocephalus octodecemspinosus* |  | 20 |  | 0.024 | 0.024 | 0.032 | 0.022 |
| *Scomber scombrus* |  | 10 |  | 0.000 | 0.000 | 0.000 | 0.000 |
| *Lophius americanus* |  | 3 |  | 0.000 | 0.000 | 0.000 | 0.000 |
| *Ammodytes dubius* |  | 71 |  | 0.422 | 0.071 | 0.400 | 0.321 |
| *Pandalus borealis* |  | 46 |  | 0.000 | 0.000 | 0.000 | 0.000 |
| *Zoarces americanus* |  | 18 |  | 0.000 | 0.000 | 0.000 | 0.000 |
| *Hippoglossoides platessoides* |  | 99 |  | 0.000 | 0.000 | 0.000 | 0.000 |
| *Pollachius pollachius* |  | 25 |  | 0.054 | 0.306 | 0.054 | 0.109 |
| *Chaceon quinquedens* |  | 14 |  | 0.000 | 0.000 | 0.000 | 0.000 |
| *Sebastes fasciatus* |  | 49 |  | 0.371 | 0.335 | 0.408 | 0.373 |
| *Urophycis chuss* |  | 7 |  | 0.000 | 0.000 | 0.000 | 0.000 |
| *Cancer irroratus* |  | 10 |  | 0.000 | 0.000 | 0.000 | 0.000 |
| *Hemitripterus americanus* |  | 6 |  | 0.000 | 0.000 | 0.000 | 0.000 |
| *Merluccius bilinearis* |  | 38 |  | 0.008 | 0.018 | 0.000 | 0.000 |
| *Malacoraja senta* |  | 5 |  | 0.000 | 0.000 | 0.000 | 0.000 |
| *Amblyraja radiata* |  | 12 |  | 0.003 | 0.033 | 0.003 | 0.019 |
| *Urophycis tenuis* |  | 46 |  | 0.015 | 0.063 | 0.000 | 0.003 |
| *Pseudopleuronectes americanus* |  | 25 |  | 0.020 | 0.046 | 0.045 | 0.051 |
| *Leucoraja ocellata* |  | 15 |  | 0.003 | 0.006 | 0.002 | 0.019 |
| *Limanda ferruginea* |  | 92 |  | 0.009 | 0.023 | 0.003 | 0.006 |
| Totals |  | 957 |  | 1.000 | 1.000 | 1.000 | 1.000 |

| Table S3. Bias and root mean squared error (RMSE) of diet estimates for adult female polar bears *Ursus maritimus* generated by each of the six QFASA variants, based on the marine mammal prey data set and computed across 500 randomly generated FASs | | | | | | | | | | | | | | | | | | | |
| --- | --- | --- | --- | --- | --- | --- | --- | --- | --- | --- | --- | --- | --- | --- | --- | --- | --- | --- | --- |
|  |  |  | Kullback-Leibler | | | | | | | |  | Aitchison | | | | | | | |
|  | Truediet |  | Prey space | |  | Pred. space | |  | Both spaces | |  | Prey space | |  | Pred. space | |  | Both spaces | |
| Prey species |  |  | Bias | RMSE |  | Bias | RMSE |  | Bias | RMSE |  | Bias | RMSE |  | Bias | RMSE |  | Bias | RMSE |
| *Erignathus barbatus* | 0.065 |  | -0.011 | 0.050 |  | -0.019 | 0.049 |  | -0.015 | 0.049 |  | -0.004 | 0.038 |  | -0.004 | 0.038 |  | -0.005 | 0.038 |
| *Delphinapterus leucas* | 0.005 |  | 0.000 | 0.011 |  | -0.002 | 0.010 |  | -0.003 | 0.006 |  | -0.000 | 0.009 |  | 0.002 | 0.014 |  | -0.001 | 0.009 |
| *Balaena mysticetus* | 0.051 |  | -0.013 | 0.026 |  | -0.003 | 0.030 |  | -0.007 | 0.027 |  | -0.009 | 0.023 |  | -0.003 | 0.024 |  | -0.005 | 0.023 |
| *Histriophoca fasciata* | 0.000 |  | 0.008 | 0.019 |  | 0.010 | 0.027 |  | 0.009 | 0.022 |  | 0.014 | 0.031 |  | 0.021 | 0.047 |  | 0.015 | 0.034 |
| *Pusa hispida* | 0.873 |  | -0.015 | 0.072 |  | -0.032 | 0.094 |  | -0.024 | 0.085 |  | -0.021 | 0.061 |  | -0.042 | 0.082 |  | -0.030 | 0.070 |
| *Phoca largha* | 0.000 |  | 0.025 | 0.051 |  | 0.035 | 0.070 |  | 0.032 | 0.064 |  | 0.016 | 0.034 |  | 0.021 | 0.046 |  | 0.020 | 0.042 |
| *Odobenus rosmarus* | 0.006 |  | 0.007 | 0.015 |  | 0.010 | 0.019 |  | 0.009 | 0.016 |  | 0.005 | 0.011 |  | 0.005 | 0.011 |  | 0.005 | 0.011 |
| Sum absolute values | 1.000 |  | 0.078 | 0.244 |  | 0.111 | 0.299 |  | 0.100 | 0.270 |  | 0.069 | 0.206 |  | 0.099 | 0.262 |  | 0.081 | 0.227 |

| Table S4. Bias and root mean squared error (RMSE) of diet estimates for female grey seals *Halichoerus grypus* sampled in the spring generated by each of the six QFASA variants, based on the marine fish and shellfish prey data set and computed across 500 randomly generated FASs | | | | | | | | | | | | | | | | | | | |
| --- | --- | --- | --- | --- | --- | --- | --- | --- | --- | --- | --- | --- | --- | --- | --- | --- | --- | --- | --- |
|  |  |  | Kullback-Leibler | | | | | | | |  | Aitchison | | | | | | | |
|  | Truediet |  | Prey space | |  | Pred. space | |  | Both spaces | |  | Prey space | |  | Pred. space | |  | Both spaces | |
| Prey species |  |  | Bias | RMSE |  | Bias | RMSE |  | Bias | RMSE |  | Bias | RMSE |  | Bias | RMSE |  | Bias | RMSE |
| *Mallotus villosus* | 0.013 |  | 0.014 | 0.029 |  | 0.017 | 0.036 |  | 0.051 | 0.060 |  | 0.012 | 0.029 |  | 0.014 | 0.032 |  | 0.019 | 0.036 |
| *Gadus morhua* | 0.005 |  | 0.021 | 0.040 |  | 0.016 | 0.033 |  | 0.012 | 0.028 |  | 0.012 | 0.031 |  | 0.010 | 0.026 |  | 0.013 | 0.031 |
| *Clupea harengus* | 0.053 |  | -0.012 | 0.031 |  | -0.008 | 0.033 |  | -0.036 | 0.042 |  | -0.001 | 0.034 |  | 0.002 | 0.037 |  | 0.002 | 0.036 |
| *Myoxocephalus octodecemspinosus* | 0.024 |  | -0.014 | 0.019 |  | -0.014 | 0.018 |  | -0.023 | 0.023 |  | -0.014 | 0.019 |  | -0.016 | 0.019 |  | -0.016 | 0.019 |
| *Ammodytes dubius* | 0.422 |  | 0.022 | 0.044 |  | -0.025 | 0.043 |  | 0.010 | 0.037 |  | 0.020 | 0.042 |  | -0.024 | 0.042 |  | -0.001 | 0.036 |
| *Pollachius pollachius* | 0.054 |  | -0.017 | 0.031 |  | -0.020 | 0.029 |  | -0.046 | 0.048 |  | -0.015 | 0.032 |  | -0.020 | 0.031 |  | -0.023 | 0.034 |
| *Sebastes fasciatus* | 0.371 |  | -0.043 | 0.052 |  | 0.016 | 0.034 |  | -0.046 | 0.057 |  | -0.044 | 0.054 |  | 0.016 | 0.038 |  | -0.024 | 0.042 |
| *Merluccius bilinearis* | 0.008 |  | 0.007 | 0.020 |  | 0.006 | 0.019 |  | 0.056 | 0.062 |  | 0.012 | 0.030 |  | 0.010 | 0.028 |  | 0.016 | 0.034 |
| *Amblyraja radiata* | 0.003 |  | -0.002 | 0.004 |  | -0.001 | 0.004 |  | -0.003 | 0.003 |  | -0.002 | 0.003 |  | -0.002 | 0.003 |  | -0.002 | 0.003 |
| *Urophycis tenuis* | 0.015 |  | 0.001 | 0.017 |  | -0.001 | 0.014 |  | -0.004 | 0.014 |  | -0.003 | 0.016 |  | -0.005 | 0.014 |  | -0.003 | 0.016 |
| *Pseudopleuronectes americanus* | 0.020 |  | -0.003 | 0.010 |  | -0.006 | 0.010 |  | -0.002 | 0.009 |  | 0.002 | 0.011 |  | -0.002 | 0.009 |  | 0.001 | 0.010 |
| *Leucoraja ocellata* | 0.003 |  | -0.002 | 0.003 |  | -0.003 | 0.003 |  | -0.003 | 0.003 |  | -0.003 | 0.003 |  | -0.003 | 0.003 |  | -0.003 | 0.003 |
| *Limanda ferruginea* | 0.009 |  | -0.002 | 0.010 |  | -0.004 | 0.009 |  | -0.009 | 0.009 |  | -0.003 | 0.010 |  | -0.004 | 0.009 |  | -0.005 | 0.008 |
| Others combined | 0.000 |  | 0.031 | 0.035 |  | 0.027 | 0.031 |  | 0.042 | 0.047 |  | 0.028 | 0.034 |  | 0.025 | 0.031 |  | 0.025 | 0.031 |
| Sum absolute values | 1.000 |  | 0.192 | 0.347 |  | 0.165 | 0.318 |  | 0.342 | 0.442 |  | 0.172 | 0.350 |  | 0.152 | 0.323 |  | 0.153 | 0.339 |

| Table S5. Bias and root mean squared error (RMSE) of diet estimates for female grey seals *Halichoerus grypus* sampled in the fall generated by each of the six QFASA variants, based on the marine fish and shellfish prey data set and computed across 500 randomly generated FASs | | | | | | | | | | | | | | | | | | | |
| --- | --- | --- | --- | --- | --- | --- | --- | --- | --- | --- | --- | --- | --- | --- | --- | --- | --- | --- | --- |
|  |  |  | Kullback-Leibler | | | | | | | |  | Aitchison | | | | | | | |
|  | Truediet |  | Prey space | |  | Pred. space | |  | Both spaces | |  | Prey space | |  | Pred. space | |  | Both spaces | |
| Prey species |  |  | Bias | RMSE |  | Bias | RMSE |  | Bias | RMSE |  | Bias | RMSE |  | Bias | RMSE |  | Bias | RMSE |
| *Mallotus villosus* | 0.022 |  | 0.007 | 0.026 |  | 0.015 | 0.036 |  | 0.039 | 0.050 |  | 0.005 | 0.025 |  | 0.007 | 0.028 |  | 0.013 | 0.030 |
| *Gadus morhua* | 0.017 |  | 0.015 | 0.042 |  | 0.010 | 0.034 |  | 0.014 | 0.038 |  | 0.001 | 0.027 |  | -0.002 | 0.023 |  | 0.002 | 0.027 |
| *Clupea harengus* | 0.014 |  | -0.001 | 0.019 |  | 0.000 | 0.021 |  | -0.012 | 0.014 |  | 0.006 | 0.024 |  | 0.007 | 0.026 |  | 0.007 | 0.026 |
| *Myoxocephalus octodecemspinosus* | 0.032 |  | -0.014 | 0.022 |  | -0.017 | 0.023 |  | -0.026 | 0.028 |  | -0.018 | 0.024 |  | -0.021 | 0.025 |  | -0.020 | 0.024 |
| *Ammodytes dubius* | 0.400 |  | 0.021 | 0.043 |  | -0.025 | 0.041 |  | 0.009 | 0.037 |  | 0.021 | 0.041 |  | -0.022 | 0.039 |  | 0.001 | 0.034 |
| *Pollachius pollachius* | 0.054 |  | -0.014 | 0.031 |  | -0.018 | 0.029 |  | -0.042 | 0.046 |  | -0.014 | 0.032 |  | -0.019 | 0.031 |  | -0.022 | 0.034 |
| *Sebastes fasciatus* | 0.408 |  | -0.046 | 0.054 |  | 0.016 | 0.034 |  | -0.049 | 0.058 |  | -0.046 | 0.055 |  | 0.019 | 0.038 |  | -0.023 | 0.040 |
| *Merluccius bilinearis* | 0.000 |  | 0.006 | 0.013 |  | 0.006 | 0.013 |  | 0.044 | 0.052 |  | 0.019 | 0.034 |  | 0.017 | 0.031 |  | 0.023 | 0.038 |
| *Amblyraja radiata* | 0.003 |  | -0.002 | 0.003 |  | -0.002 | 0.003 |  | -0.003 | 0.003 |  | -0.002 | 0.003 |  | -0.003 | 0.003 |  | -0.002 | 0.003 |
| *Urophycis tenuis* | 0.000 |  | 0.005 | 0.010 |  | 0.003 | 0.008 |  | 0.003 | 0.007 |  | 0.003 | 0.007 |  | 0.002 | 0.006 |  | 0.003 | 0.009 |
| *Pseudopleuronectes americanus* | 0.045 |  | -0.003 | 0.011 |  | -0.011 | 0.014 |  | -0.008 | 0.013 |  | 0.005 | 0.012 |  | -0.005 | 0.010 |  | 0.000 | 0.010 |
| *Leucoraja ocellata* | 0.002 |  | -0.002 | 0.002 |  | -0.002 | 0.002 |  | -0.002 | 0.002 |  | -0.002 | 0.002 |  | -0.002 | 0.002 |  | -0.002 | 0.002 |
| *Limanda ferruginea* | 0.003 |  | 0.001 | 0.008 |  | -0.001 | 0.005 |  | -0.003 | 0.003 |  | 0.000 | 0.006 |  | -0.001 | 0.005 |  | -0.002 | 0.004 |
| Others combined | 0.000 |  | 0.029 | 0.033 |  | 0.025 | 0.030 |  | 0.036 | 0.041 |  | 0.025 | 0.033 |  | 0.022 | 0.029 |  | 0.022 | 0.030 |
| Sum absolute values | 1.000 |  | 0.167 | 0.318 |  | 0.150 | 0.293 |  | 0.290 | 0.392 |  | 0.166 | 0.326 |  | 0.148 | 0.297 |  | 0.142 | 0.310 |

| Table S6. Bias and root mean squared error (RMSE) of diet estimates for male grey seals *Halichoerus grypus* sampled in the fall generated by each of the six QFASA variants, based on the marine mammal prey data set and computed across 500 randomly generated FASs | | | | | | | | | | | | | | | | | | | |
| --- | --- | --- | --- | --- | --- | --- | --- | --- | --- | --- | --- | --- | --- | --- | --- | --- | --- | --- | --- |
|  |  |  | Kullback-Leibler | | | | | | | |  | Aitchison | | | | | | | |
|  | Truediet |  | Prey space | |  | Pred. space | |  | Both spaces | |  | Prey space | |  | Pred. space | |  | Both spaces | |
| Prey species |  |  | Bias | RMSE |  | Bias | RMSE |  | Bias | RMSE |  | Bias | RMSE |  | Bias | RMSE |  | Bias | RMSE |
| *Mallotus villosus* | 0.030 |  | 0.011 | 0.030 |  | 0.021 | 0.041 |  | 0.064 | 0.071 |  | 0.004 | 0.022 |  | 0.007 | 0.025 |  | 0.013 | 0.027 |
| *Gadus morhua* | 0.010 |  | 0.011 | 0.031 |  | 0.009 | 0.027 |  | 0.009 | 0.029 |  | 0.009 | 0.029 |  | 0.006 | 0.024 |  | 0.013 | 0.031 |
| *Clupea harengus* | 0.037 |  | -0.009 | 0.028 |  | -0.007 | 0.029 |  | -0.032 | 0.034 |  | 0.006 | 0.029 |  | 0.010 | 0.032 |  | 0.009 | 0.031 |
| *Myoxocephalus octodecemspinosus* | 0.022 |  | -0.012 | 0.018 |  | -0.012 | 0.018 |  | -0.021 | 0.022 |  | -0.010 | 0.018 |  | -0.012 | 0.017 |  | -0.012 | 0.017 |
| *Ammodytes dubius* | 0.321 |  | 0.013 | 0.038 |  | -0.021 | 0.038 |  | 0.012 | 0.037 |  | 0.014 | 0.038 |  | -0.017 | 0.037 |  | 0.006 | 0.034 |
| *Pollachius pollachius* | 0.109 |  | -0.013 | 0.030 |  | -0.023 | 0.033 |  | -0.070 | 0.074 |  | -0.018 | 0.036 |  | -0.029 | 0.040 |  | -0.034 | 0.045 |
| *Sebastes fasciatus* | 0.373 |  | -0.044 | 0.051 |  | 0.015 | 0.032 |  | -0.067 | 0.072 |  | -0.048 | 0.055 |  | 0.013 | 0.032 |  | -0.031 | 0.043 |
| *Merluccius bilinearis* | 0.000 |  | 0.010 | 0.019 |  | 0.008 | 0.016 |  | 0.082 | 0.087 |  | 0.011 | 0.023 |  | 0.011 | 0.021 |  | 0.018 | 0.030 |
| *Amblyraja radiata* | 0.019 |  | -0.007 | 0.013 |  | -0.007 | 0.013 |  | -0.012 | 0.015 |  | -0.006 | 0.012 |  | -0.008 | 0.012 |  | -0.003 | 0.010 |
| *Urophycis tenuis* | 0.003 |  | 0.011 | 0.020 |  | 0.009 | 0.016 |  | 0.013 | 0.021 |  | 0.004 | 0.013 |  | 0.003 | 0.011 |  | 0.007 | 0.015 |
| *Pseudopleuronectes americanus* | 0.051 |  | 0.001 | 0.011 |  | -0.009 | 0.013 |  | 0.000 | 0.010 |  | 0.005 | 0.012 |  | -0.005 | 0.011 |  | -0.001 | 0.010 |
| *Leucoraja ocellata* | 0.019 |  | -0.012 | 0.015 |  | -0.014 | 0.016 |  | -0.019 | 0.019 |  | -0.015 | 0.016 |  | -0.015 | 0.017 |  | -0.017 | 0.018 |
| *Limanda ferruginea* | 0.006 |  | -0.001 | 0.009 |  | -0.001 | 0.008 |  | -0.006 | 0.006 |  | 0.000 | 0.010 |  | -0.001 | 0.008 |  | -0.002 | 0.007 |
| Others combined | 0.000 |  | 0.040 | 0.045 |  | 0.034 | 0.039 |  | 0.046 | 0.050 |  | 0.043 | 0.048 |  | 0.038 | 0.042 |  | 0.036 | 0.040 |
| Sum absolute values | 1.000 |  | 0.195 | 0.357 |  | 0.191 | 0.338 |  | 0.453 | 0.546 |  | 0.194 | 0.360 |  | 0.175 | 0.328 |  | 0.202 | 0.359 |


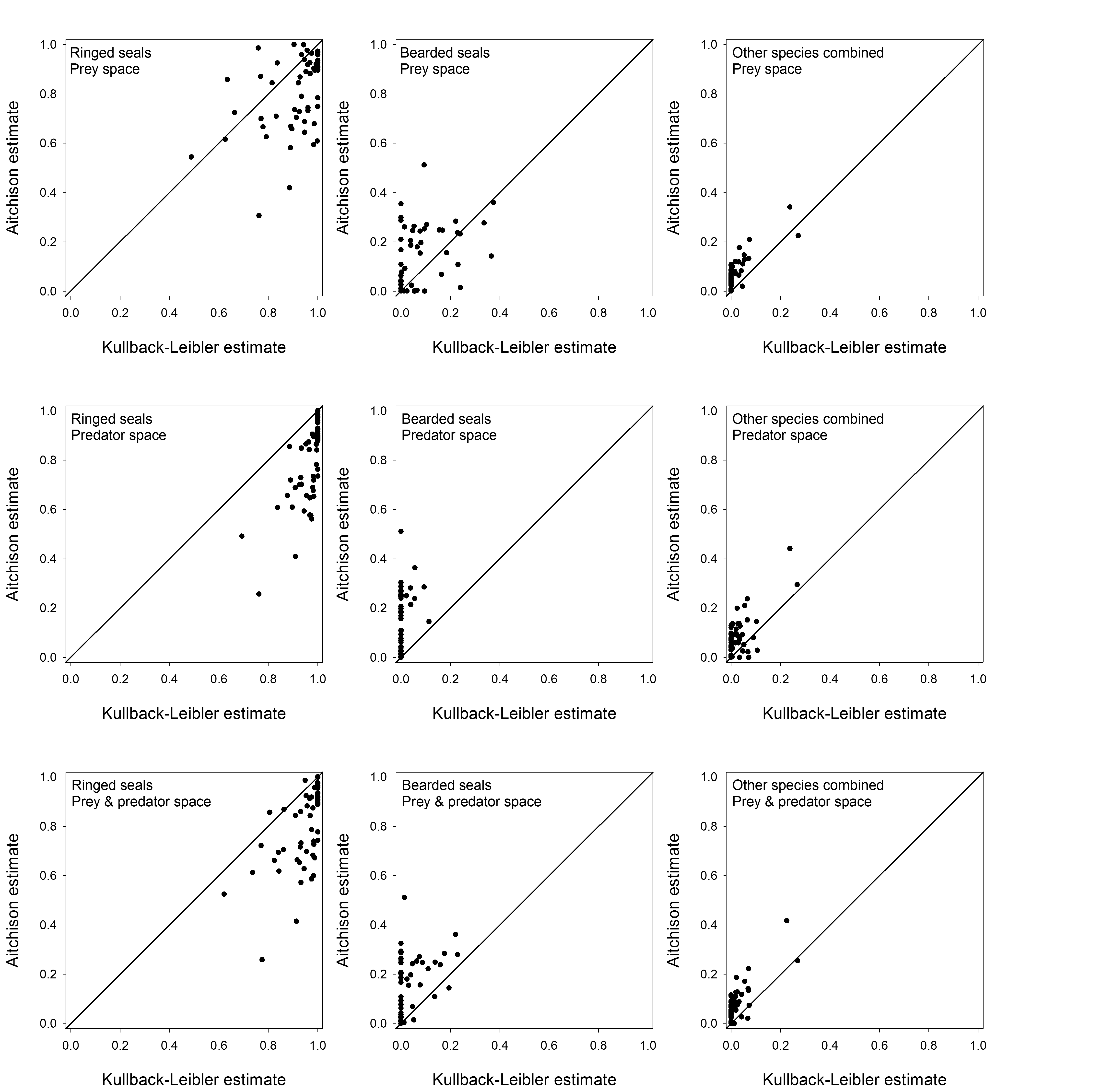


Fig. S1 . Estimated contributions (proportions) of ringed seal *Pusa hispida*, bearded seal *Erignathus barbatus*, and all other species combined to the diets of individual adult female Chukchi Sea polar bears *Ursus maritimus*. The mean diets of these bears were previously reported by Rode et al. (2014).


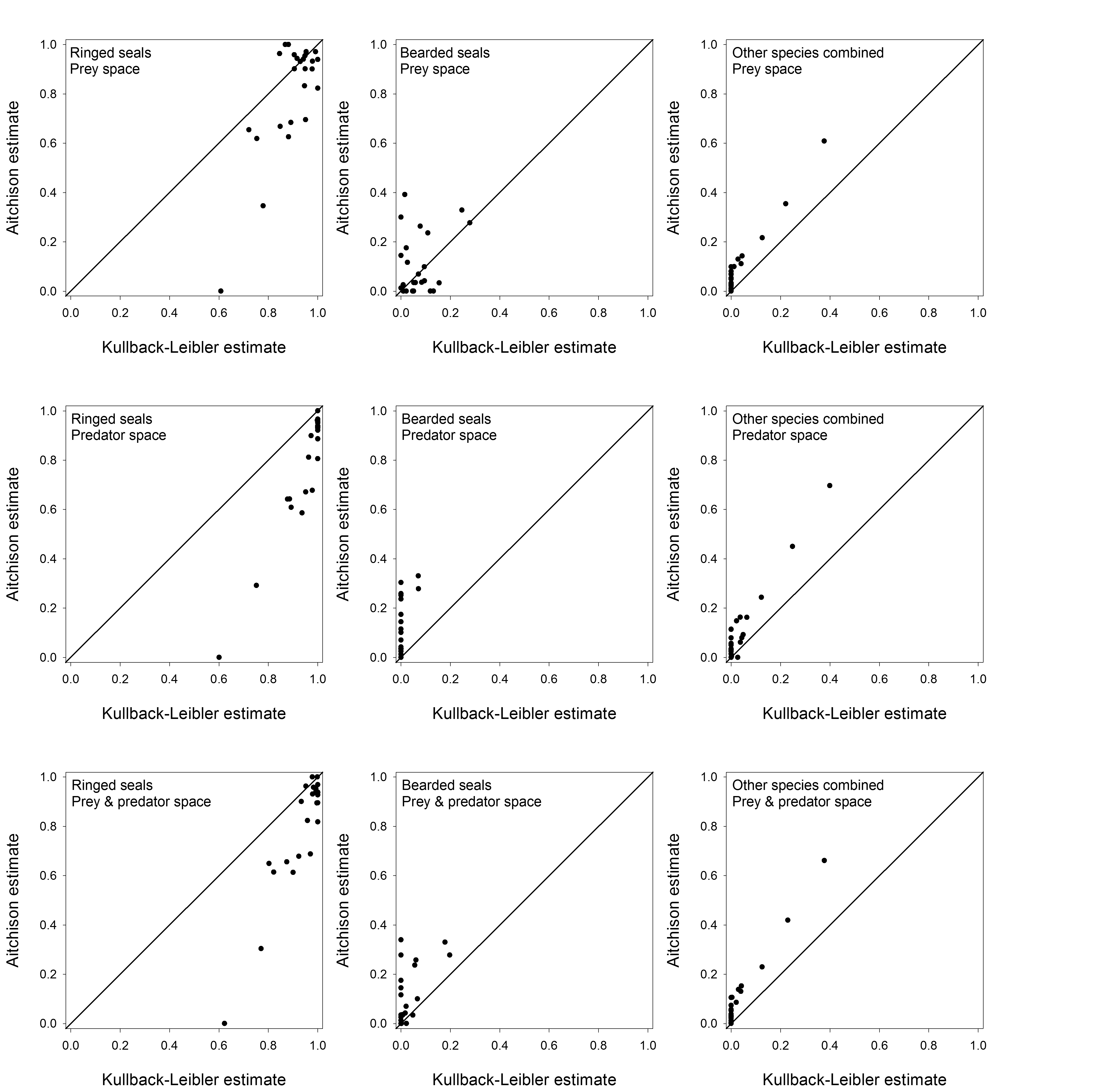


Fig. S2 . Estimated contributions (proportions) of ringed seal *Pusa hispida*, bearded seal *Erignathus barbatus*, and all other species combined to the diets of individual subadult male Chukchi Sea polar bears *Ursus maritimus*. The mean diets of these bears were previously reported by Rode et al. (2014).


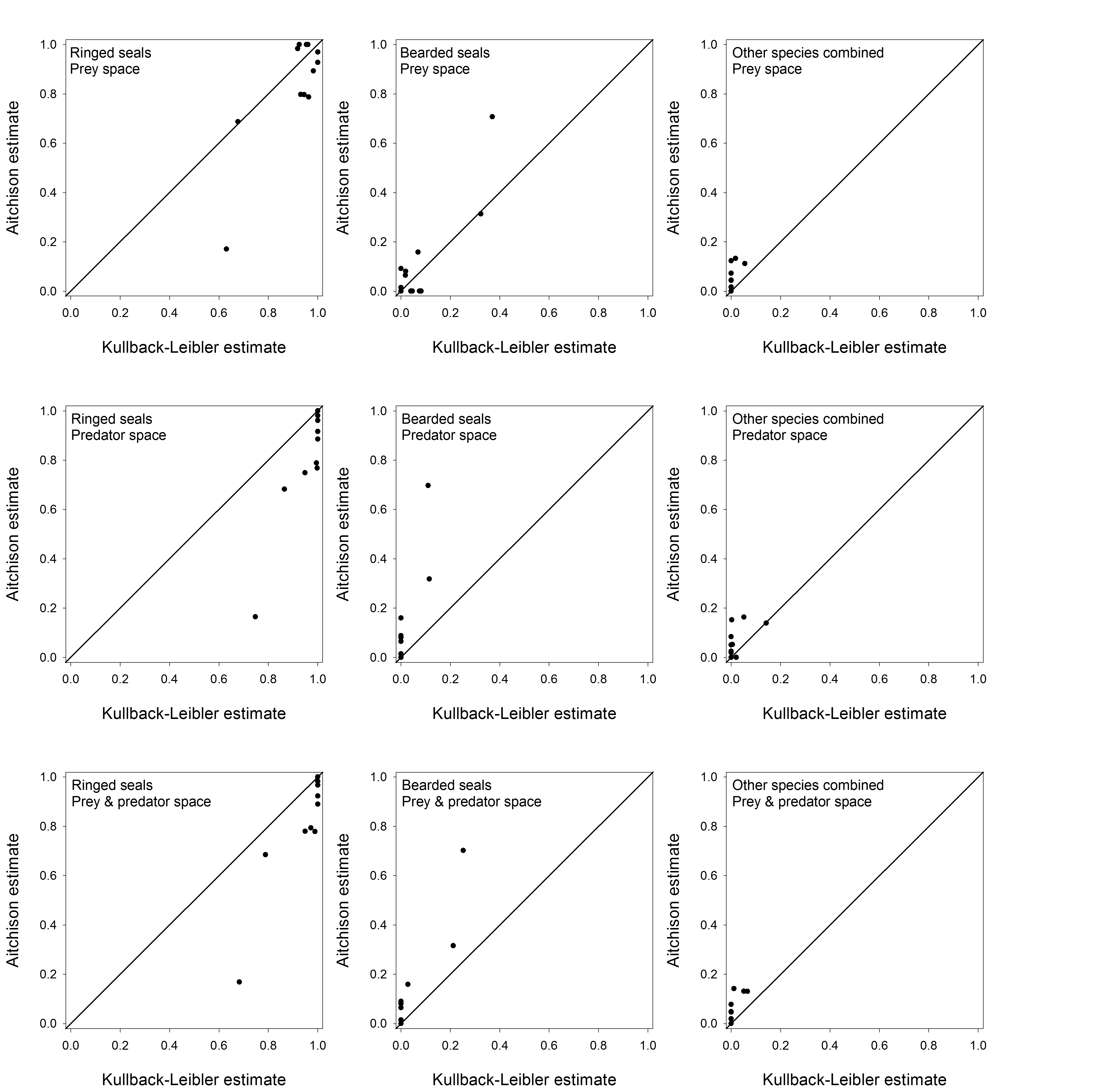


Fig. S3 . Estimated contributions (proportions) of ringed seal *Pusa hispida*, bearded seal *Erignathus barbatus*, and all other species combined to the diets of individual subadult female Chukchi Sea polar bears *Ursus maritimus*. The mean diets of these bears were previously reported by Rode et al. (2014).


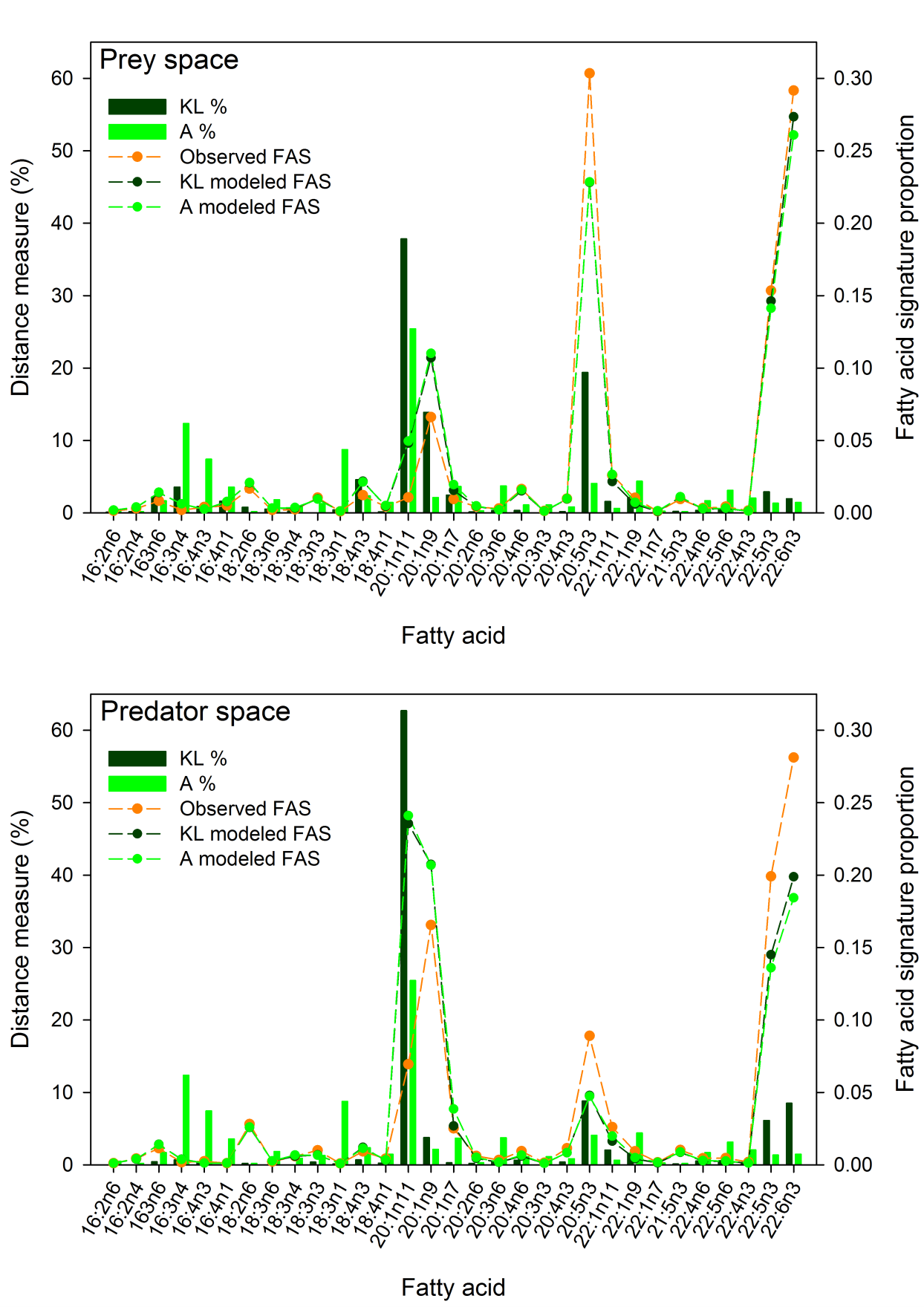


Fig. S4. The percentages of the Kullback-Leibler and Aitchison distance measures attributable to each fatty acid (bars) and the observed and modeled fatty acid signatures (lines) in the prey (top panel) and predator (bottom panel) estimation spaces, averaged over all adult female Chukchi Sea polar bears *Ursus maritimus*.


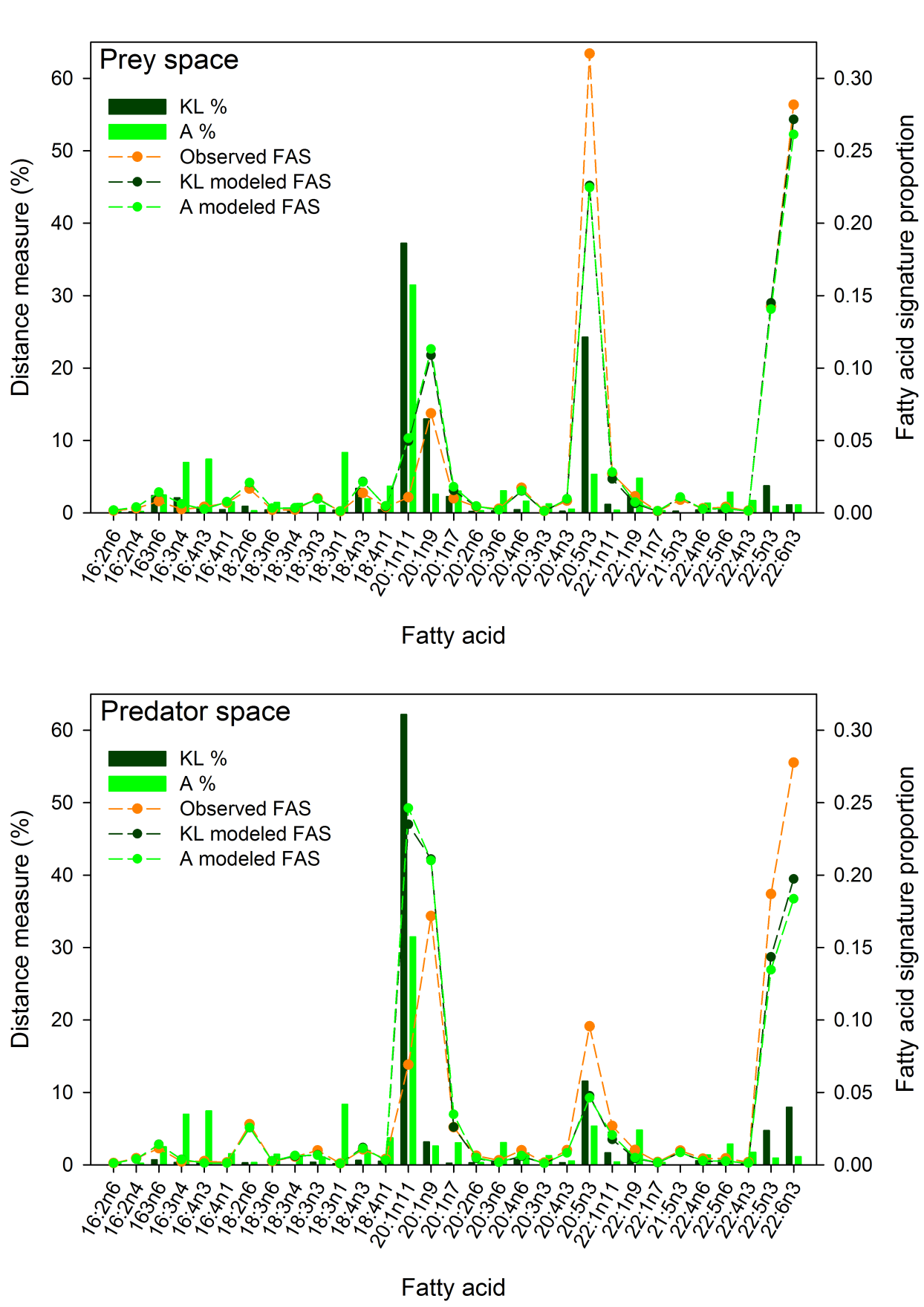


Fig. S5. The percentages of the Kullback-Leibler and Aitchison distance measures attributable to each fatty acid (bars) and the observed and modeled fatty acid signatures (lines) in the prey (top panel) and predator (bottom panel) estimation spaces, averaged over all subadult male Chukchi Sea polar bears *Ursus maritimus*.


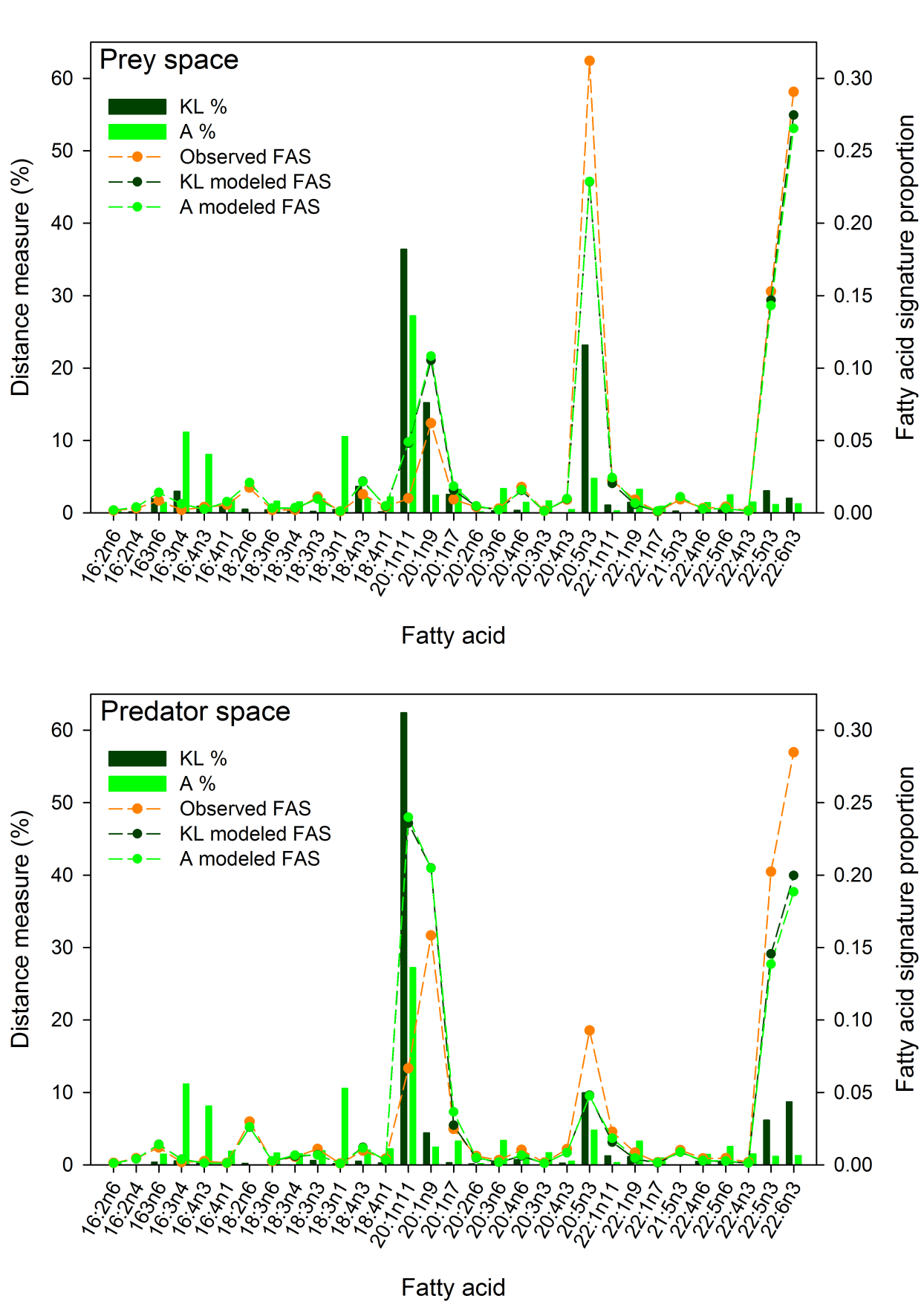


Fig. S6. The percentages of the Kullback-Leibler and Aitchison distance measures attributable to each fatty acid (bars) and the observed and modeled fatty acid signatures (lines) in the prey (top panel) and predator (bottom panel) estimation spaces, averaged over all subadult female Chukchi Sea polar bears *Ursus maritimus*.
